# High iodine promotes autoimmune thyroid disease by activating hexokinase 3 and inducing polarization of macrophages towards M1

**DOI:** 10.3389/fimmu.2022.1009932

**Published:** 2022-10-17

**Authors:** Tiantian Cai, Peng Du, Lixia Suo, Xiaozhen Jiang, Qiu Qin, Ronghua Song, Xiaorong Yang, Yanfei Jiang, Jin-an Zhang

**Affiliations:** ^1^ Department of Endocrinology & Rheumatology, Shanghai University of Medicine & Health Sciences Affiliated Zhoupu Hospital, Shanghai, China; ^2^ Department of Endocrinology, Shanghai University of Medicine & Health Sciences Affiliated Jiading District Central Hospital, Shanghai, China; ^3^ Department of Endocrinology, Shanghai Pudong New Area People’s Hospital, Shanghai, China

**Keywords:** autoimmune thyroid disease, graves’ disease, hashimoto’s thyroiditis, macrophage polarization, iodine, hexokinase 3

## Abstract

Autoimmune thyroid disease (AITD), the most common autoimmune disease, includes Graves’ disease (GD) and Hashimoto’s thyroiditis (HT). Currently, the pathogenesis of AITD is not fully understood. Our study aimed to examine the presence of macrophage polarization imbalance in AITD patients, to investigate whether high iodine can cause macrophage polarization imbalance, and to investigate the role of key genes of metabolic reprogramming in macrophage polarization imbalance caused by high iodine. We synergistically used various research strategies such as systems biology, clinical studies, cell culture and mouse disease models. Gene set enrichment analysis (GSEA) revealed that M1 macrophage hyperpolarization was involved in the pathogenesis of AITD. *In vitro* and *in vivo* experiments showed that high iodine can affect the polarization of M1 or M2 macrophages and their related cytokines. Robust rank aggregation (RRA) method revealed that hexokinase 3 (HK3) was the most aberrantly expressed metabolic gene in autoimmune diseases. *In vitro* and *in vivo* studies revealed HK3 could mediate macrophage polarization induced by high iodine. In summary, hyperpolarization of M1-type macrophages is closely related to the pathogenesis of AITD. High iodine can increase HK3 expression in macrophages and promote macrophage polarization towards M1. Targeting HK3 can inhibit M1 polarization induced by high iodine.

## Introduction

Autoimmune thyroid disease (AITD) is an organ-specific autoimmune disease and the most common autoimmune disease in the world. Both Graves’ disease (GD) and Hashimoto’s thyroiditis (HT) are its common types (1, 2). Although the clinical symptoms of GD and HT are obviously different, they share some common pathogenic mechanisms, such as large amounts of lymphocyte infiltration in thyroid accompanied by thyroid tissue destruction. At present, the treatment of AITD is still mainly symptomatic therapy, and there is still no effective immunotargeted therapy for the etiology of AITD, so it is necessary to search for effective immunotherapy strategies through in-depth study on the pathogenesis of the disease.

*In vivo* immune homeostasis is a necessary condition for maintaining autoimmune tolerance and inhibiting autoimmune injury (3, 4). Immune cells such as antigen presenting cells (APCs), T cells and B cells all play key regulatory roles in the maintenance of immune homeostasis *in vivo *(5, 6). Excessive activation or abnormal function of macrophages, T cells, and B cells can cause imbalance of immune homeostasis *in vivo* and further trigger autoimmune diseases (7, 8). Current studies have proved that adaptive immune cells such as T cells and B cells play an important role in the pathogenesis of AITD, and the overactivation of these cells is closely related to the occurrence of AITD (9, 10). However, there is no systematic study on the role of macrophage in the pathogenesis of AITD. Macrophages are important phagocytes and APCs in the body. The main function of macrophages is to phagocytose danger signals such as cell fragments and pathogens in the body, present antigens to adaptive immune cells, and further activate T cells and B cells to produce subsequent immune responses (11, 12). Macrophage polarization refers to the differentiation of macrophages in phenotype and function induced by various factors such as microenvironment and inflammatory factors. Macrophages can be divided into two types according to their function and cytokines secreted, namely, M1 macrophages with pro-inflammatory and pro-immune response functions and M2 macrophages with anti-inflammatory and repair functions (13). Current studies believe that the overactivation of M1 macrophages can mistakenly present autoantigens to T cells and B cells, causing the body to produce autoantibodies or autoreactive lymphocytes, thus triggering autoimmune diseases. At the same time, M1 macrophages can also mediate the occurrence of autoimmune diseases by producing various proinflammatory factors (14). Several studies have shown that abnormal polarization of macrophages is closely related to the occurrence and progression of various autoimmune diseases, including rheumatoid arthritis (RA), inflammatory bowel disease (IBD), systemic lupus erythematosus (SLE), type 1 diabetes mellitus (T1DM), and other autoimmune diseases (15–17).

Iodine is the most important environmental factor affecting the occurrence of thyroid diseases. In our previous population-based and cross-sectional study, we found a U-shaped relationship between adult iodine intake and thyroid autoimmunity. Iodine deficiency, especially excess iodine, is a risk factor for AITD in adults (18). Existing studies believe that high iodine may trigger AITD through various mechanisms (19, 20). Recent studies have found that iodine can directly promote the secretion of cytokines in human peripheral blood lymphocytes (21), and iodine transmembrane transporters such as sodium iodide symporter (NIS) transporters are also expressed in monocyte-macrophages to a certain extent. These findings suggest that iodine may directly activate or regulate the immune function of monocyte-macrophages. Metabolic reprogramming is changes in energy metabolism of immune cells during differentiation or activation (22). Up to now, many studies have found that metabolic reprogramming of the macrophages is closely related to the occurrence of autoimmune diseases and cancers, and the key molecules associated with metabolic reprogramming are expected to become the novel targets in the treatment of these diseases (23–27). Changes in the internal environment, such as hypoxia, nutrition, danger signals, and cytokines, can regulate the phenotype and function of macrophages by influencing the metabolic reprogramming (28–30).

Therefore, based on the above findings, we hypothesize that high iodine intake may cause an imbalance in the polarization of macrophages through their metabolic reprogramming, which leads to AITD. Our study aims to examine the presence of macrophage polarization imbalance in thyroid tissue and peripheral blood of AITD patients, to investigate whether high iodine can cause macrophage polarization imbalance, and to investigate the role of key genes of metabolic reprogramming in macrophage polarization imbalance caused by high iodine through various research strategies.

## Materials and methods

### Transcriptome profiling datasets of AITD thyroid tissue and peripheral CD14^+^ monocyte-macrophages

Four GD thyroid tissue and three normal thyroid specimens were obtained from patients undergoing surgical treatment in Shanghai University of Medicine & Health Sciences Affiliated Zhoupu Hospital. RNA extracted from tissue was used for NimbleGen Human Microarray Chip (Roche, USA) to detect transcriptome data from each sample. The gene expression values were extracted and standardized using NimbleScan v2.5 software. In addition, two AITD thyroid tissue transcriptome microarray data series (GSE29315 and GSE6339) in Gene Expression Omnibus (GEO) database were downloaded and annotated as the gene expression matrix using the corresponding annotation document of the platform. GSE29315 was used for HT research, while GD microarray data of our study was used for GD research. GSE6339 was used for AITD research (that is, HT and GD were taken as a whole object).

Furthermore, we collected CD14^+^ monocyte-macrophages samples from 19 GD, 10 HT and 11 sex- and age-matched healthy controls. After peripheral blood mononuclear cells (PBMCs) were separated by density gradient centrifugation, CD14^+^ monocyte-macrophages were sorted by human CD14 magnetic beads (Miltenyi Biotec, No.130-050-201). Total RNA of these cells was extracted for mRNA-sequencing (mRNA-seq) using BGISEQ-500 sequencing platform. The obtained raw sequencing data were aligned to human reference genome and the gene expression values were calculated.

### Establishment of macrophage polarization characteristic gene set and Gene Set Enrichment Analysis

In this study, the transcriptome datasets related to macrophage polarization were integrated by Robust rank aggregation (RRA) method (31) to establish a set of characteristic genes for macrophage polarization. First, we searched the GEO database for genome-wide transcriptome data related to macrophage polarization. In order to obtain objective and accurate results, only M1 macrophage polarization data series induced by IFN-γ or lipopolysaccharide (LPS) and M2 macrophage polarization data series induced by IL-4 were included in this study. Moreover, the included array data series had at least 3 replicates in each group, and the sequencing data series had at least 2 replicates in each group. Then, we made a RRA analysis to establish a characteristic gene set of macrophage polarization. We first calculated the differentially expressed genes (DEGs) of each dataset using “limma” or “DESeq2” package, and then generated the gene ranking matrix according to Fold change (FC), and finally used “Robust Rank Aggregation” package to complete integration analysis. After that, we selected the top 500 up-ranked genes in the RRA integration analysis as preliminary characteristic genes for M1 or M2 polarization. After further excluding the intersection of the two gene sets, the remaining genes were selected as the final M1 polarization or M2 polarization characteristic gene set. In order to verify the reliability of the above macrophage polarization characteristic gene set, we adopted other data series (GSE82227, GSE123603 and GSE123180) related to macrophage polarization for verification by Gene Set Enrichment Analysis (GSEA) (32, 33). We also used GSEA to study the enrichment of M1 and M2 polarization characteristic gene sets in AITD thyroid tissue and peripheral CD14^+^ monocyte-macrophages and to evaluate whether the imbalance of macrophage polarization was closely related to AITD.

### Immunohistochemical analysis

Immunohistochemistry was used to analyze the infiltration of macrophages and the expression of hexokinase 3 (HK3) in AITD thyroid tissue. CD68 antibody (NB100-683, Novusbio), secondary antibody (K4001, DAKO), HK3 antibody (13333-1-AP, Proteintech) and secondary antibody (K4003, DAKO) were used for immunohistochemical analysis of CD68 and HK3 expression in the thyroid tissue sections of AITD and controls. After antigen retrieval and removal of the blocking solution, each section was incubated for 1 h with 100 μL of diluted primary antibodies and then with 100 μL of secondary antibody for 30 min at room temperature. The positive cells were revealed by incubating with 100 μL of freshly prepared chromogenic solution.

### Cell culture

RAW264.7 cells (murine monocyte-macrophages line) and human PBMCs were used to investigate *in vitro* whether high iodine could cause macrophage polarization imbalance. RAW264.7 cells were cultured in Roswell Park Memorial Institute (RPMI) 1640 medium containing 10% fetal bovine serum (FBS) and 1% penicillin and streptomycin. RAW264.7 cells were inoculated into 6-well plates at 2×10^5^/mL, with three replicates in each group. The high iodine solution was prepared by sodium iodide (NaI, No.7681-82-5, Adamas). To evaluate the effect of high iodine on the polarization of macrophages, RAW264.7 cells were stimulated with NaI at 0 mM, 0.1 mM, 1 mM and 10 mM concentrations. Supernatant and cells were collected after 48 h of stimulation.

A total of 20 mL of heparin sodium anticoagulant peripheral blood samples were collected from a healthy control. PBMCs were divided into 9 equal portions and cultured in RPMI 1640 complete medium for 48 h under the intervention conditions of 0 mM, 1 mM and 10 mM of NaI (3 replicates per group). Supernatant and cells were also collected after stimulation.

In subsequent studies, to dynamically observe the effect of HK3 on macrophage polarization, RAW264.7 cells were transfected with HK3-specific short hairpin RNA (shRNA) lentivirus and negative lentivirus vector and stimulated with 0 mM and 10 mM NaI. After 48 h of intervention, cells were collected for flow cytometry.

### Cytokine detection of supernatant

Supernatant of RAW264.7 cells and PBMCs was used for cytokine detection. The protein expression levels of IL-6 and IL-1β in the supernatant of RAW264.7 cell culture were determined by Enzyme-Linked immunosorbent assay (ELISA) kits (IL-6: M6000B, R&D and IL-1β: DY401, R&D). The protein expression levels of IL-6 and IL-1β in the supernatant of human PBMCs culture were measured using Cytometric Bead Array (CBA) method. The magnetic beads were used to detect IL-6 (No. 558276, BD) and IL-1β (No. 558279, BD).

### Quantitative real-time PCR

RNA was extracted from cells by Trizol method and reverse transcribed into a cDNA library. Quantitative real-time PCR (qRT-PCR) was performed using SYBR Green PCR Master Mix with a total reaction volume of 15 μL on the Applied Biosystems QuantStudio 7 Flex system. The PCR procedure was as follows: one cycle at 95°C for 15 seconds, followed by 45 two-step cycles, specifically 95°C for 5 seconds and 63°C for 34 seconds. The internal reference of mouse samples was Gapdh, and that of human samples was ACTB. Primer sequences are shown in **Table S1**.

### Preparation of thyroiditis model in mice

In this study, a mouse thyroiditis model was constructed by high iodine water feeding and thyroglobulin immuno-injection, which was described in our previous study in detail (34). Female 6–8 weeks old C57BL/6 mice from Shanghai Model Organisms Center were kept at the SPF level and free to eat and drink. Thirty-five mice were randomly divided into two groups. One was the thyroiditis model group (n=20) and the other was the control group (n=15). The model group was fed with high iodine water (0.05%NaI), and the control group was fed with ordinary drinking water. The whole animal model preparation time was 6 weeks. At the end of the experiment, the mice were sacrificed and their spleens were ground into cell suspension, which was used for flow cytometry to analyze the proportion of spleen macrophages.

In the subsequent study, to further study the mechanism of HK3 *in vivo*, ten female wild-type mice and ten female HK3-knockout homozygous mice were used for preparation of thyroiditis models (named as WH and KH group, respectively). Their spleens tissue was taken for flow cytometry and thyroid tissue were used for mRNA-seq.

### Flow cytometry analysis

The proportion of M1 (CD45^+^CD11b^+^Ly6G^-^F4/80^+^CD206^-^) and M2 (CD45^+^CD11b^+^Ly6G^-^F4/80^+^CD206^+^) in RAW264.7 cells and mouse spleen macrophages were analyzed by flow cytometry. In brief, cells samples were labeled with surface antibodies CD45 (APC-Cy7, 565853, BD), CD11b (PE-Cy7, 101216, Biolegend), Ly6G (BV421, 562737, BD) and F4/80(Alexa Fluor, 565853, BD) and incubated at 4°C for 20 min away from light. After that, the cells were incubated with lysis buffer at 4°C for 20 min away from light and then with antibody CD206 (PE, 141706, Biolegend) for 30 min in the dark for intracellular staining. The major subsets of monocyte-macrophages in human circulation (CD14^++^CD16^-^, CD14^+^CD16^+^, CD14^+^CD68^+^CCR2^+^, and CD14^+^CD163^+^CX3CR1^+^) were also detected by flow cytometry. Similarly, PBMCs were collected after the primary culture and then incubated with surface antibodies CD14 (APC-Cy7, 557831, BD), CD16 (PerCP-Cy5.5, 560717, BD), CCR2 (Alexa Fluor-647, 8018933, BD), CD163 (PE-Cy7, 556018, BD), and CX3CR1 (PE, 565796, BD), as well as intracellular staining antibody CD68 (FITC, IC20401F, RD) in sequence. These cells were resuspended with 200 μL of stain buffer, and then detected by CytoFLEX LX flow cytometer (Beckman Coulter).

### Autoimmune disease transcriptome datasets and RRA analysis

In order to screen for abnormal expressions of key genes of metabolic reprogramming in autoimmune diseases, we used RRA method to systematically study the expressions of 402 key metabolic genes in peripheral blood of patients with autoimmune diseases (**Table S2**). We searched the peripheral blood genome-wide expression profile datasets for autoimmune diseases from GEO database. The inclusion criteria for the datasets were as follows: 1) The diseases studied were common autoimmune diseases; 2) The tissue used were whole blood or PBMCs; 3) Genome-wide mRNA expression levels were analyzed by microarray or mRNA-seq; 4) More than 50 DEGs were found; and 5) more than 90% of the 402 key metabolic genes were studied in this study.

### The role of HK3 in GD by systems biology analyses

We used systems biology tools to further analyze the role of HK3 in the pathogenesis of GD. First, CIBERSORT was used to analyze the proportion of M1 and M2 macrophages in 18 cases of thyroid tissue in GSE9340 (35), and to analyze their correlations with HK3. Secondly, we studied whether HK3 was related to the imbalance of macrophage polarization in GD tissue by using GSEA method. Finally, weighted gene co-expression network analysis (WGCNA) was used to analyze the key HK3-related co-expression gene modules in GD thyroid tissue, and gene ontology (GO) was used to analyze the molecular function of co-expression modules (36).

### Statistical analysis

Continuous variables were expressed as mean ± SE. The Mann-Whitney U test or t test was used to compare differences between groups. Statistical analysis was performed using STATA (12.0, StataCorp, USA), and a two-sided P value <0.05 indicated a statistically significant difference. The GSEA software 3.0 (http://software.broadinstitute.org/gsea/index.jsp) was used in the analysis. Enrichment score (ES), nominal P value, and false discovery rate (FDR) q value were calculated. Conventionally, a gene set with an ES value greater than 0.5 and FDR q value less than 0.25 was deemed as a significantly enriched gene set. Data of microarray and mRNA-seq were analyzed using R software (version 3.5.1).

## Results

### M1 polarization gene set was significantly enriched in thyroid tissue and peripheral blood CD14+ monocyte-macrophages of AITD patients

By searching the GEO database and assessing quality, 118 transcriptome microarray or sequencing datasets on macrophage polarization were included in this study. We completed the integrated analysis of the DEGs in the above datasets using the RRA tool. **Tables S3–S6** show datasets related to M1 and M2 macrophage polarization in human and mouse, respectively. **Table S7** and **Table S8** respectively shows the M1 polarization and M2 polarization characteristic gene sets of human and mouse macrophages established in this study. To investigate the representativeness of the above gene sets, we analyzed M1 or M2 polarization in GSE82227, GSE123603, and GSE123180 by GSEA method, and found that the gene sets of macrophage polarization established in our study had significant reliability (**Figure S1**).

**Figure 1:**
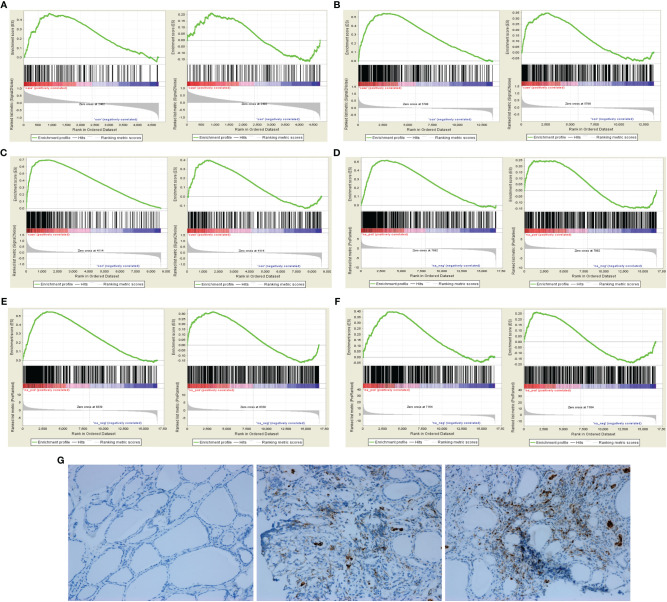
GSEA of macrophage polarization and immunohistochemical analysis in AITD patients. **(A)** In AITD thyroid tissue (From left to right: M1 and M2); **(B)** In GD thyroid tissue (From left to right: M1 and M2); **(C)** In HT thyroid tissue (From left to right: M1 and M2); **(D)** In CD14^+^ monocyte-macrophages of AITD patients (From left to right: M1 and M2); **(E)** In CD14^+^ monocyte-macrophages of GD patients (From left to right: M1 and M2); **(F)** In CD14^+^ monocyte-macrophages of HT patients (From left to right: M1 and M2); **(G)** Immunohistochemical results of CD68 in controls, HT and GD patients in sequence.

In GSEA analysis of AITD, the results showed that M1 polarization gene set was enriched in AITD thyroid tissue but without significance (ES=0.47, P=0.02, FDR q=0.02). In GSEA analysis of GD and HT, the results showed that M1 polarization gene set was significantly enriched in GD thyroid tissue (ES=0.54, P<0.001, FDR q=0.12) and HT thyroid tissue (ES=0.70, P=0.04, FDR q=0.13), but not for M2 polarization gene set (**Figures 1A–C**). In GSEA analysis of peripheral CD14^+^ monocyte-macrophages of AITD patients, M1 polarization gene set was significantly enriched in CD14+ monocyte-macrophages of patients with AITD and GD (AITD: ES=0.52, P<0.001, FDR q<0.001; GD: ES=0.55, P<0.001, FDR q<0.001), but not for M2 polarization gene set. HT patients showed a moderate enrichment of M1 polarization gene set in the CD14+ monocyte-macrophages (ES=0.40, P <0.001, FDR q <0.001) (**Figures 1D–F**). In addition, immunohistochemical results indicated that macrophage marker molecule CD68 was significantly expressed in both GD and HT thyroid tissue, but not in normal thyroid tissue (**Figure 1G**).

### High iodine could cause an imbalance of macrophage polarization *in vitro* and *in vivo* studies

**Figure 2:**
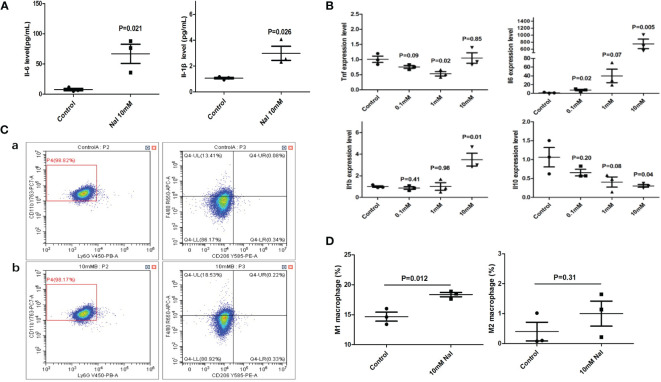
High iodine stimulation of RAW264.7. **(A)** ELISA results of supernatants; **(B)** qRT-PCR results; **(C)** Flow cytometry of macrophage proportions. a: 0 mM of NaI control group; b:10 mM of NaI intervention group; **(D)** Statistics of macrophage proportions.

**Figure 3:**
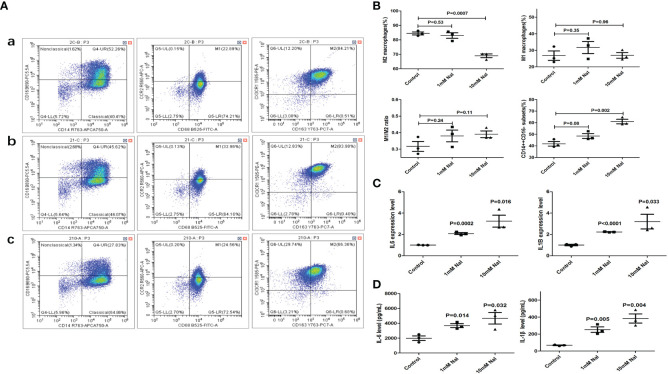
High iodine stimulation of human PBMCs. **(A)** Flow cytometry of macrophage proportions. a: 0 mM of NaI control group; b:1mM of NaI intervention group; c: 10mM of NaI intervention group; **(B)** Statistics of macrophage proportions (10mM NaI could significantly reduce the proportion of M2 macrophages and increase the proportion of pro-inflammatory monocytes); **(C)** qRT-PCR results; **(D)** CBA results of supernatants.

After high iodine stimulation of RAW264.7, ELISA results confirmed that the expression levels of M1 characteristic molecules IL-1 β and IL-6 increased after high iodine (10mM of NaI) stimulation (P=0.026 and 0.021, respectively) (**Figure 2A**). qRT-PCR further showed that the mRNA expression levels of IL-1β and IL-6 increased after high iodine (10mM of NaI) stimulation (P=0.01 and 0.005, respectively), while the expression levels of M2 characteristic molecule IL-10 decreased (P=0.04) (**Figure 2B**). Results of flow cytometry showed that high iodine (10 mM of NaI) induced excessive polarization of macrophages to M1 and increased the proportion of M1 macrophages (14.7% vs 18.4%, P=0.012). There was no significant effect on the proportion of M2 macrophages (0.4% vs 1.0%, P=0.31) (**Figures 2C, D**). Human PBMCs were also stimulated by high iodine. It was found that high iodine (10 mM of NaI) could induce the transformation of monocytes into the pro-inflammatory type (CD14^++^CD16^-^) (41.8% vs 61.0%, P=0.002) and significantly reduce the proportion of M2 macrophages (CD14^+^CD163^+^CX3CR1^+^) (84.4% vs 68.9%, P=0.0007). However, high iodine stimulation had no effect on the proportion of M1 macrophages (CD14^+^CD68^+^CCR2^+^) and M1/M2 ratio (**Figures 3A, B**). Results of PCR and CBA also confirmed that high iodine could promote the expressions of IL-6 and IL1-β in PBMCs (P<0.05, **Figures 3C, D**).

**Figure 4:**
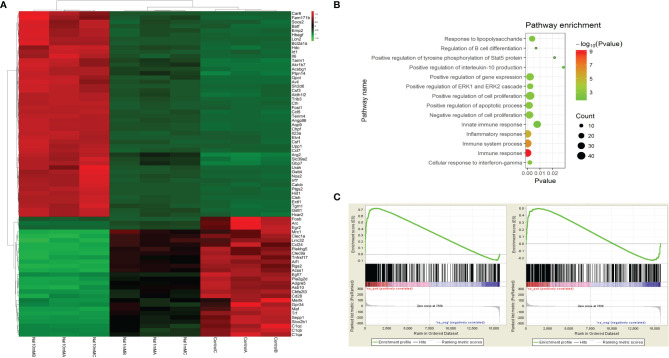
mRNA-seq of RAW264.7 cells stimulated by high iodine. **(A)** Differences in the mRNA transcriptome of various groups (NaI of 0 mM, 1mM and 10mM); **(B)** GO analysis; **(C)** GSEA of macrophage polarization in transcriptome of RAW264.7 cells stimulated by 10 mM of NaI (From left to right: M1 and M2).

To further study the effect of high iodine on the polarization of macrophages, we systematically studied the transcriptome profile of RAW264.7 cells stimulated by high iodine. The mRNA-seq results showed that 10 mM of NaI could significantly promote the polarization of macrophages and increase the expression levels of pro-inflammatory and pro-immune response molecules. NaI of 1 mM had a certain activation effect on macrophages and increased the expression levels of some key immune molecules, but the effect was weaker than 10 mM of NaI (**Figure 4A**), while 0.1 mM of NaI had no significant effect on the activation of macrophages. GO analysis found that high iodine could activate several immune response-related pathways in macrophages, such as immune response, innate immune response, IFN-γ pathway, and other signaling pathways (**Figure 4B**). GSEA further showed that 0.1 mM and 1 mM of NaI did not significantly affect macrophage polarization, while 10 mM of NaI significantly promoted M1 polarization of macrophages (ES=0.71, P<0.001, FDR q<0.001), and had no remarkable effect on M2 polarization of macrophages. (**Figure 4C**).

**Figure 5:**
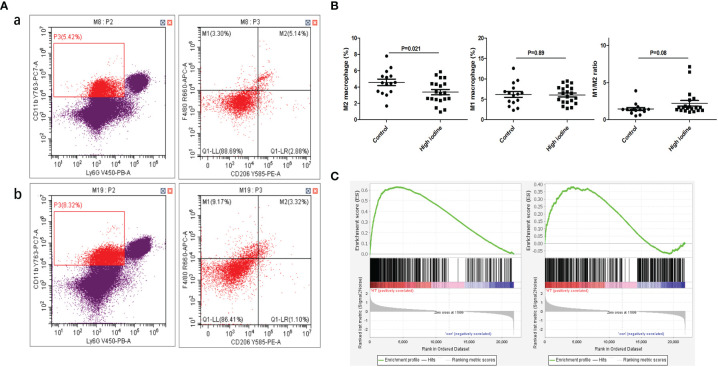
Effect of high iodine on mouse macrophage polarization. **(A)** Flow cytometry of macrophage proportions in spleen tissue. a: control group; b: model group; **(B)** Statistics of macrophage proportions; **(C)** GSEA of macrophage polarization in transcriptome of thyroid tissue from model group (From left to right: M1 and M2).

In the experiment of mouse thyroiditis model, we analyzed the effect of high iodine on the polarization of mouse spleen macrophages by flow cytometry. It was found that the proportion of M2 macrophages (CD45^+^CD11b^+^Ly6G^-^F4/80^+^CD206^+^) in the model group was significantly decreased when compared with control group (4.6% vs 3.4%, P=0.021). There was no significant effect on M1 macrophages (CD45^+^CD11b^+^Ly6G^-^F4/80^+^CD206^-^) (6.1% vs 6.2%, P=0.89). High iodine had a certain promotion in M1/M2 ratio, but there was no statistically significant difference (1.4 vs 2.2, P=0.08) (**Figures 5A, B**). Besides, we also performed GSEA analysis of macrophage polarization in mouse thyroid tissue. mRNA-seq data were obtained from our previous study (34), including normal controls (n=3) and thyroiditis model group (n=5). GSEA results showed that M1 polarization characteristic gene set was significantly enriched in the thyroiditis group (ES=0.63, P<0.001, FDR q<0.001), while M2 was not (ES=0.38, P<0.001, FDR q<0.001) (**Figure 5C**).

### HK3 was highly expressed in PBMCs and thyroid tissue of AITD

**Figure 6:**
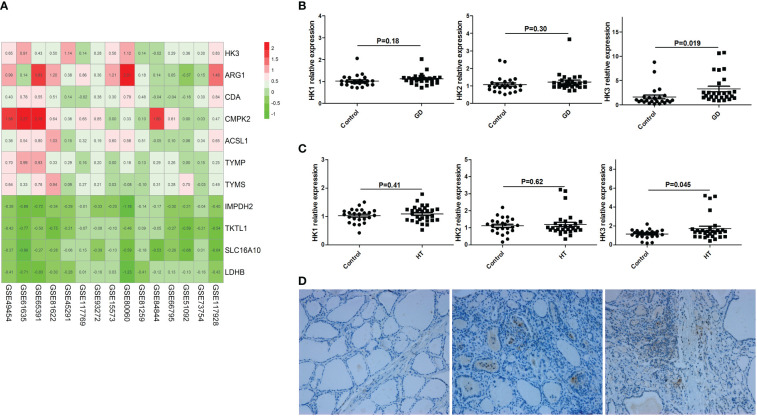
HK3 was highly expressed in PBMCs and thyroid tissue of AITD. **(A)** RRA analysis (HK3 was the most significant key metabolic gene); **(B)** mRNA expression of HK1, HK2 and HK3 in PBMCs of GD patients; **(C)** mRNA expression of HK1, HK2 and HK3 in PBMCs of HT patients; **(D)** Immunohistochemical results of HK3 in controls, HT and GD patients in sequence.

By searching GEO database, we found 15 datasets of peripheral blood transcriptome profiles of patients with autoimmune diseases that met the inclusion criteria of our study (**Table S9**). We used RRA analysis to evaluate the expressions of 402 key metabolic genes in these patients with autoimmune diseases, and found that HK3 was the most significant key metabolic gene with abnormal expression in autoimmune diseases (adjusted P=1.2×10^-9^, **Figure 6A**). We further verified the expression of HK3 in PBMCs and thyroid tissue of AITD patients. qRT-PCR results showed that the expression level of HK3 in PBMCs of GD (n=30) and HT patients (n=30) was significantly higher than that of controls (n=30) (P=0.019 and 0.045, respectively), while there was no abnormal expression of HK1 and HK2 in PBMCs of AITD patients (P>0.05) (**Figures 6B, C**). Immunohistochemical studies showed that HK3 was significantly expressed in both GD and HT thyroid tissue, but not in normal thyroid tissue (**Figure 6D**).

### High iodine could promote HK3 expression in macrophages and knockdown of HK3 could significantly inhibit the degree of M1 polarization

**Figure 7:**
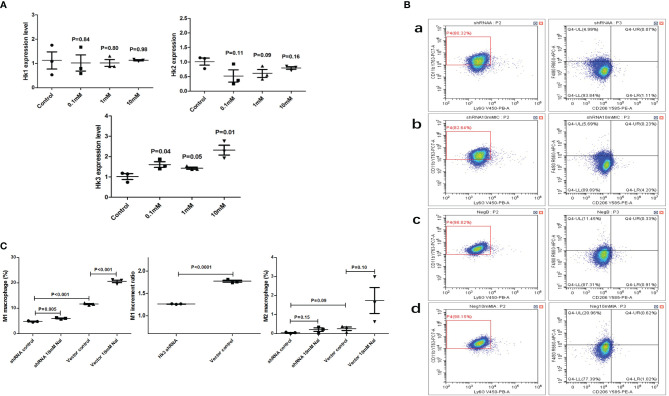
High iodine can promote HK3 expression in RAW264.7 cells. **(A)** mRNA expression of HK1, HK2 and HK3 after high iodine stimulation; **(B)** Flow cytometry of macrophage proportions. a: HK3 shRNA lentivirus-control group; b: HK3 shRNA lentivirus-high iodine stimulation group; c: negative lentivirus-control group; d: negative lentivirus high-iodine stimulation group; **(C)** Statistics of macrophage proportions.

*In vitro* cell culture, it was found that high iodine (10 mM of NaI) stimulation could increase the expression level of HK3 in RAW264.7 cells (P=0.01), but had no significant effect on HK1 and HK2 (P>0.05) (**Figure 7A**). We further transfected RAW264.7 cells with HK3 shRNA lentivirus to knock down HK3 expression level. The proportion of M1 macrophages in the HK3 shRNA lentivirus-transfected group was increased by 1.26-fold after high iodine (10 Mm of NaI) stimulation (4.8% vs 6.0%, P=0.005). The proportion of M1 macrophages was also significantly increased by 1.77-fold (11.6% vs 20.6%, P<0.001) in the negative lentivirus transfection group after high iodine (10 mM of NaI) stimulation. Compared with the negative lentivirus group, the M1 macrophages increment ratio decreased significantly in the HK3 shRNA lentivirus transfected group (1.77-fold vs 1.26-fold, P<0.001), indicating that targeted knockdown of HK3 expression level in macrophages can significantly inhibit the degree of M1 polarization of macrophages induced by high iodine (**Figures 7B, C**).

### HK3 gene knockout could cause an imbalance of macrophage polarization in spleen and thyroid tissue

**Figure 8:**
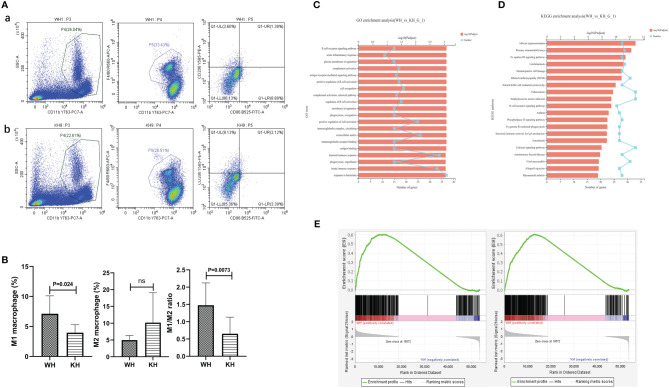
*In vivo* experiment of HK3 gene knockout mice. **(A)** Flow cytometry of macrophage proportions in spleen tissue. a: WH group; b:KH group; **(B)** Statistics of macrophage proportions; **(C)** GO analysis; **(D)** KEGG analysis; **(E)** GSEA of macrophage polarization in transcriptome of thyroid tissue from WH group (From left to right: M1 and M2).

A total of 20 female C57BL/6 mice were used in this study. Mouse spleens from WH and KH group were ground and the cells were examined by flow cytometry. The results showed that compared with WH group, the proportion of M1 macrophages and the ratio of M1/M2 were significantly decreased (5.74% vs 3.93%, P=0.024; 1.48 vs 0.65, P=0.007, respectively), suggesting that HK3 knockout inhibited M1 polarization of macrophages in mouse spleen (**Figures 8A, B**). mRNA-seq of thyroid tissue was used to screen out DEGs between the two groups (5 mice per group). GO analysis revealed that DEGs were involved in biological processes such as response to bacterium, innate immune response, humoral immune response and positive regulation of cell activation (**Figure 8C**). KEGG analysis found that DEGs were enriched in a variety of immune-related pathways, including natural killer cell-mediated cytotoxicity pathway, B cell receptor signaling pathway, Fcγ R-mediated phagocytosis, and AITD (**Figure 8D**). GSEA results showed that both M1 and M2 polarization signature gene sets were significantly enriched in the thyroid tissue of WH group (M1: ES=0.60, P<0.001, FDR q<0.001; M2: ES=0.61, P<0.001, FDR q<0.001). In other words, M1 and M2 were significantly polarized in the wild-type thyroiditis group compared with the HK3- knockout thyroiditis group (**Figure 8E**).

### Systems biology revealed the role of HK3 in the pathogenesis of GD

According to the data of The Human Protein Atlas (www.proteinatlas.org), HK3 is mainly expressed in immune-related tissue, especially in monocyte-macrophages. In addition, according to the mRNA-seq data of CD14^+^ monocyte-macrophages, the expression abundance of HK3 in CD14^+^ monocyte-macrophages were also significantly higher than that of HK1 and HK2 (**Figure S2A**), suggesting that HK3 may be the main glycolysis enzyme playing a role in monocyte-macrophages.

Furthermore, we analyzed the proportion of major immune cells in the GSE9340 dataset using CIBERSORT (**Figure S2B**). It was found that HK3 was significantly correlated with the M1 macrophages proportion and M1/M2 ratio in GD thyroid tissue (**Figure S2C**). In WGCNA analysis of GD thyroid tissue, the Darkred module was most correlated with HK3, with a correlation coefficient of 0.85 (P=1.0×10^-5^) (**Figures S2D, E**). Key genes in the co-expression module of Darkred mainly included CXCL9, CXCL10, CD84, IFNG, TNF, IFI30, TYROBP, TLR8 and other genes (**Figure S2F**). GO enrichment analysis found that the function of this module was mainly enriched in various immune pathways (**Figure S2G**). GSEA analysis showed that HK3 in GD thyroid tissue was significantly related to the M1 macrophage polarization (ES=0.66, P=0.002, FDR q=0.002), but not to M2 polarization (ES=0.42, P=0.02, FDR q=0.09) (**Figure S2H**). These results suggest that HK3 may be involved in the pathogenesis of GD by affecting the polarization of macrophages.

## Discussion

Macrophage polarization is one of the research hotspots in recent years, and it plays a key role in the pathogenesis of many diseases (37–39). By studying the role of macrophage polarization and its key regulatory factors in the pathogenesis of some diseases, it is expected to provide new therapeutic strategies or targets for those diseases (15). Currently, there is little research on the role of macrophage polarization in the pathogenesis of AITD. Therefore, in order to study the role of macrophage polarization in AITD, it is imperative to conduct an in-depth analysis from a holistic and systematic perspective (40–42). In this study, a set of characteristic genes for macrophage polarization was established using systems biology methods. In addition, we further studied the role of macrophage polarization in AITD by GSEA analysis. The results showed that M1 polarization gene set were significantly enriched in thyroid tissue and peripheral blood CD14^+^ monocyte-macrophages of patients with GD and HT, suggesting that M1 polarization is closely related to the occurrence and progression of AITD. Immunohistochemical study also showed that macrophage infiltration was evident in both GD and HT thyroid tissue. These results demonstrated the important role of innate immune pathways in the pathogenesis of AITD from the perspective of macrophage polarization. In consequence, the targeted regulation of macrophage polarization is expected to become a new strategy for the treatment of AITD.

Current studies have found that genetic, environmental, immune and other factors can affect the function of macrophages, induce their polarization imbalance, and further trigger a variety of immune-related diseases (43–46). A range of environmental factors, such as high-salt diet and vitamin D deficiency, can promote the transformation of macrophages into the pro-inflammatory type, thus affecting the balance of macrophage polarization and promoting the occurrence of immune-related diseases (47–50). Binger et al. showed that high salt could inhibit the polarization of M2 macrophages, leading to the imbalance of M1/M2 ratio and increasing the risk of autoimmune diseases (48). Another study showed that a high-salt diet could activate macrophages in mice and increase the expression of pro-inflammatory and immune-related genes (47). In this study, we proposed the hypothesis that high iodine, as an important environmental risk factor for AITD, could induce the imbalance of macrophage polarization and promote the occurrence of AITD. In the present study, we have confirmed that high iodine can promote the unbalanced polarization of macrophages through various methods. First, we stimulated mouse monocyte-macrophages with high iodine *in vitro* and found that high iodine could significantly promote M1 polarization of macrophages. Then, we conducted primary culture of human PBMCs *in vitro* and found that high iodine could inhibit M2 macrophage polarization and induce the transformation of monocytes into the pro-inflammatory type. Finally, we used the thyroiditis model to further confirm that high iodine caused imbalance of macrophage polarization *in vivo*. From multiple perspectives, our study established that iodine could induce M1 polarization or inhibit M2 polarization, resulting in the imbalance of macrophage polarization and ultimately promoting the occurrence of AITD. Besides, in order to further study the molecular mechanism involved in the high-iodine-mediated imbalance of macrophage polarization, we studied the transcriptome expression profile of macrophages stimulated by high iodine through mRNA-seq, and found that DEGs were associated with several immune response-related pathways. These results suggest that high iodine may affect macrophage polarization through a variety of immunological mechanisms, and more studies are needed for further investigations.

It is important to note that despite the above findings, several issues need to be highlighted in the study of the effects of high iodine on macrophage polarization. 1) Although a concentration gradient of NaI was set to stimulate RAW264.7 cells and PBMCs *in vitro*, the results showed that only 10mM of NaI significantly induced macrophage polarization. Based on the studies of others (21), we hypothesized that under the stimulation of low concentration of NaI, macrophages could balance the iodine concentration inside and outside the cells through the down-regulation of NIS expression, and maintain their own homeostasis without leading to obvious polarization. However, in response to high iodine stimulation, this homeostasis may be broken and macrophage polarization occurs. 2) Although the qPCR and CBA results of human PBMCs stimulated by high iodine *in vitro* showed that IL-6 and IL-1β were significantly upregulated, these two cytokines, which may be derived from lymphocytes, could not directly explain the effect of high iodine on macrophage polarization and cytokine production, but only as indirect evidence. 3) The polarization of macrophages seemed to be different in RAW264.7 cells, PBMCs, and tissue of thyroiditis model mice. In our study, hyperpolarization of M1 mainly occurred in RAW264.7 cells *in vitro* and thyroid tissue *in vivo*, while in PBMCs *in vitro* and spleen tissue *in vivo*, unbalanced polarization of macrophages was triggered mainly by inhibiting M2 polarization. However, these two effects ultimately lead to the same outcome, that is, a relative increase in M1 macrophage polarization and enhanced pro-inflammatory and pro-immune effects. There are several possible explanations for the inconsistent regulation of macrophage polarization by high iodine. First of all, the immune microenvironment of macrophages *in vivo* and *in vitro* is completely different. Similarly, monocyte-macrophages from RAW264.7 cells and PBMCs, as well as macrophages in thyroid and spleen tissue, were also exposed to different immune microenvironments, which may have impact on macrophage polarization state. Secondly, the time and concentration of high iodine stimulation differed *in vivo* and *in vitro* experiments; therefore, dissimilar effects might be caused. Finally, the data of flow cytometry and GSEA could only reflect the proportion of macrophages, but could not accurately reflect the function of macrophages. In subsequent studies, more research methods could be used to accurately assess the polarization state of macrophages.

In recent years, studies have found that energy metabolism in immune cells is closely related to the function of them, which mainly includes glycolysis, tricarboxylic acid cycle, phosphopentose pathway (PPP), fatty acid oxidation, fatty acid synthesis, and amino acid metabolism (51). Metabolic reprogramming is changes in energy metabolism of immune cells during differentiation or activation (22). It has been confirmed that M1 macrophages are mainly powered by glycolysis, while M2 macrophages are mainly powered by mitochondrial oxidative phosphorylation. During M1 polarization, macrophages switch energy metabolism to glycolysis through metabolic reprogramming. ATP can be efficiently supplied to macrophages when glycolysis levels are significantly elevated. This is called the Warburg effect (also known as aerobic glycolysis). This transformation can significantly increase proliferation and pro-inflammatory and pro-immune response effects of macrophages (52). Aerobic glycolysis in macrophages can generate ribose through PPP for nucleotide synthesis, and the synthesis of NAPDH can further generate reactive oxygen species (ROS) to participate in the immune response. Key glycolytic enzymes of macrophages are important in regulating metabolic reprogramming, such as fructose-6-phosphate kinase (PFKFB3) and pyruvate kinase isoenzyme (PKM2). Dysfunction of these key enzyme can cause imbalance of macrophage polarization, and the effects of metabolic reprogramming on immune cells function mediated by different isozyme are significantly different (53–57). In addition to PFKFB3 and PKM2, hexokinase (HK) is a key enzyme in glycolysis and plays an important role in regulating energy metabolism of immune cells (58–60). Unlike the first two key enzymes of glycolysis, HK catalyzes the first step of glycolysis metabolism, and the catalyzed production of glucose 6-phosphate (G-6-P) can not only be used for glycolysis, but also enter multiple alternative pathways such as PPP pathway. Thus HK-mediated metabolic reprogramming is significantly different from PFKFB3 and PKM2 (61). There are mainly four isoenzymes of HK in humans, namely, HK1, HK2, HK3 and glucokinase (GCK, also known as HK4). At present, the research on regulation of energy metabolism by HK in cells mostly involves tumors. Targeted inhibition of HK1 or HK2 can inhibit the aerobic glycolysis of tumor cells, promote mitochondrial oxidative phosphorylation, and inhibit the proliferation and growth of tumor cells, which is a new strategy for tumor treatment (62–64). That HK3 is mainly expressed in monocyte-macrophages of the immune system proves that HK3 may play a key role in the activation or polarization of macrophages. In this study, we explored the mechanisms of macrophage polarization induced by high iodine from the perspective of HK3 through integrating systems biology, clinical studies, cell culture and animal studies. Results showed that HK3 was significantly overexpressed in thyroid tissue and peripheral PBMCs of AITD. *In vitro* studies also showed increased HK3 expression in macrophages stimulated by high iodine. In addition, both *in vivo* and *in vitro* experiments confirmed that the proportion of M1 macrophages decreased after HK3 knockdown and knockout. mRNA-seq of thyroid tissue also revealed that HK3 was involved in multiple immunity events. Meanwhile, GSEA analysis of thyroid tissue showed that M1 and M2 in the wild-type model group were significantly polarized compared with the HK3 knockout model group. These results suggest that HK3 knockout affects macrophage polarization in thyroid tissue. However, it is not clear whether the effect is greater for M1 or M2, and therefore the M1/M2 ratio cannot be derived. Taken together, as a key enzyme in glycolysis, HK3 overexpression induced by high iodine may cause hyperpolarization of macrophages to pro-inflammatory M1 type through metabolic reprogramming, which may over-activate the immune system and break the immune homeostasis in the microenvironment of organ tissue, and finally trigger AITD. The targeted regulation of HK3 to control macrophage polarization is expected to be a new approach to effective treatment of autoimmune diseases such as AITD.

There are deficiencies in this study. Due to the difficulty of collecting thyroid tissue from AITD patients and the small amount of thyroid tissue from animal models, flow cytometry cannot be used for detecting the proportion of macrophage in thyroid tissue. At the same time, the currently available high-throughput data on thyroid tissue are relatively limited, and it is not possible to use more systems biology tools to systematically and comprehensively study the pathogenesis of AITD. Subsequent in-depth studies can be conducted by collecting large samples of thyroid tissue in AITD patients, which is expected to fully reveal the pathogenesis of this disease and find effective targets for prevention and treatment.

## Data availability statement

The original contributions presented in the study are included in the article/**Supplementary Materials**. Further inquiries can be directed to the corresponding authors.

## Ethics statement

The studies involving human participants were reviewed and approved by Ethics Committee of Zhoupu Hospital of Shanghai University of Medicine & Health Sciences. The patients/participants provided their written informed consent to participate in this study. The animal study was reviewed and approved by Ethics Committee of Scientific Research of Shanghai University of Medicine & Health Sciences.

## Author contributions

TC: Conceptualization, Methodology, Data Curation, Formal Analysis, Visualization, Writing - Original Draft, Writing - Review & Editing; PD: Conceptualization, Methodology, Software, Formal Analysis, Visualization, Writing - Original Draft, Writing - Review & Editing; LS: Methodology, Visualization; XJ: Software, Validation; QQ: Resources, Supervision, Writing - Review & Editing; RS: Resources, Supervision, Visualization, Writing - Review & Editing; XY: Resources, Software, Visualization; YJ: Conceptualization, Methodology, Resources, Supervision, Project Administration, Writing - Review & Editing; JZ: Conceptualization, Funding Acquisition, Resources, Supervision, Project Administration, Writing - Review & Editing. All authors contributed to the article and approved the submitted version.

## Funding

This research was supported by The National Natural Science Foundation of China (Grant No. 81873636), Pudong New Area Health Commission key sub-specialty (PWZy2020-12), Clinical Research Center of thyroid diseases of Shanghai Health Medical College(20MC20200002), and Project of Shanghai Medical Key Specialty (ZK2019C09).

## Acknowledgments

The authors thank all the patients and volunteers who participated in this study.

## Conflict of interest

The authors declare that the research was conducted in the absence of any commercial or financial relationships that could be construed as a potential conflict of interest.

## Publisher’s note

All claims expressed in this article are solely those of the authors and do not necessarily represent those of their affiliated organizations, or those of the publisher, the editors and the reviewers. Any product that may be evaluated in this article, or claim that may be made by its manufacturer, is not guaranteed or endorsed by the publisher.

## Supplementary material

Supplementary Figure 1 GSEA validation of human and mouse macrophage polarization gene sets. **(A)** Enrichment plots of human macrophage polarization gene sets in the GSEA analysis of human M1 polarization induced by IFN-γ in GSE82227 (From left to right: M1 and M2; M1: ES=0.92 (P<0.001, FDR q <0.001; M2: ES=0.40, P=0.91, FDR q=0.91); **(B)** Enrichment plots of human macrophage polarization gene sets in the GSEA analysis of human M2 polarization induced by IL-4 in GSE123603(From left to right: M1 and M2; M1: ES=0.31, P=0.67, FDR q=0.68; M2: ES=0.93, P<0.001, FDR q <0.001); **(C)** Enrichment plots of mice macrophage polarization gene sets in the GSEA analysis of mice M1 polarization induced by LPS in GSE123180(From left to right: M1 and M2; M1:ES=0.89,P<0.001, FDR q <0.001; M2: ES=0.35, P=0.36, FDR q=0.37); **(D)** Enrichment plots of mice macrophage polarization gene sets in the GSEA analysis of mice M2 polarization induced by IL-4 in GSE123180 (From left to right: M1 and M2; M1: ES= -0.62, P<0.001, FDR q <0.001;M2: ES=0.92,P<0.001, FDR q <0.001).

Supplementary Figure 2 Systems biology revealed the role of HK3 in the pathogenesis of GD. **(A)** HK3 expression abundance in CD14^+^ monocyte-macrophages; **(B)** The proportion of major immune cells in 18 thyroid tissue in the GSE9340 dataset; **(C)** The relationship between HK3 and the proportion of M1 macrophages and M1/M2 ratio; **(D)** HK3-related co-expression gene modules by WGCNA analysis; **(E) **Darkred module was most correlated with HK3; **(F)** Heatmap of key genes of Darkred module; **(G) **GO analysis; **(H)** GSEA analysis.

## References

[1] Antonelli A Ferrari SM Corrado A Di Domenicantonio A Fallahi P. Autoimmune thyroid disorders. Autoimmun Rev (2015) 14:174–80. doi: 10.1016/j.autrev.2014.10.016 25461470

[2] Smith TJ Hegedüs L. Graves’ disease. N Engl J Med (2016) 375:1552–65. doi: 10.1056/NEJMra1510030 27797318

[3] Petersen F Yue X Riemekasten G Yu X. Dysregulated homeostasis of target tissues or autoantigens - A novel principle in autoimmunity. Autoimmun Rev (2017) 16:602–11. doi: 10.1016/j.autrev.2017.04.006 28411168

[4] Cabral-Marques O Marques A Giil LM De Vito R Rademacher J Günther J. GPCR-specific autoantibody signatures are associated with physiological and pathological immune homeostasis. Nat Commun (2018) 9:5224. doi: 10.1038/s41467-018-07598-9 30523250 PMC6283882

[5] Pereira LMS Gomes STM Ishak R Vallinoto ACR. Regulatory T cell and forkhead box protein 3 as modulators of immune homeostasis. Front Immunol (2017) 8:605. doi: 10.3389/fimmu.2017.00605 28603524 PMC5445144

[6] Chen Y Li C Lu Y Zhuang H Gu W Liu B. IL-10-Producing CD1d(hi)CD5(+) regulatory b cells may play a critical role in modulating immune homeostasis in silicosis patients. Front Immunol (2017) 8:110. doi: 10.3389/fimmu.2017.00110 28243231 PMC5303715

[7] Trahtemberg U Mevorach D. Apoptotic cells induced signaling for immune homeostasis in macrophages and dendritic cells. Front Immunol (2017) 8:1356. doi: 10.3389/fimmu.2017.01356 29118755 PMC5661053

[8] Kuchroo VK Ohashi PS Sartor RB Vinuesa CG. Dysregulation of immune homeostasis in autoimmune diseases. Nat Med (2012) 18:42–7. doi: 10.1038/nm.2621 22227671

[9] Li Q Wang B Mu K Zhang JA. The pathogenesis of thyroid autoimmune diseases: New T lymphocytes - cytokines circuits beyond the Th1-Th2 paradigm. J Cell Physiol (2019) 234:2204–16. doi: 10.1002/jcp.27180 30246383

[10] Smith MJ Rihanek M Coleman BM Gottlieb PA Sarapura VD Cambier JC. Activation of thyroid antigen-reactive b cells in recent onset autoimmune thyroid disease patients. J Autoimmun (2018) 89:82–9. doi: 10.1016/j.jaut.2017.12.001 PMC5902436 29233566

[11] Amit I Winter DR Jung S. The role of the local environment and epigenetics in shaping macrophage identity and their effect on tissue homeostasis. Nat Immunol (2016) 17:18–25. doi: 10.1038/ni.3325 26681458

[12] Luo B Gan W Liu Z Shen Z Wang J Shi R. Erythropoeitin signaling in macrophages promotes dying cell clearance and immune tolerance. Immunity (2016) 44:287–302. doi: 10.1016/j.immuni.2016.01.002 26872696

[13] Murray PJ. Macrophage polarization. Annu Rev Physiol (2017) 79:541–66. doi: 10.1146/annurev-physiol-022516-034339 27813830

[14] Funes SC Rios M Escobar-Vera J Kalergis AM. Implications of macrophage polarization in autoimmunity. Immunology (2018) 154:186–95. doi: 10.1111/imm.12910 PMC5980179 29455468

[15] Udalova IA Mantovani A Feldmann M. Macrophage heterogeneity in the context of rheumatoid arthritis. Nat Rev Rheumatol (2016) 12:472–85. doi: 10.1038/nrrheum.2016.91 27383913

[16] Tsai F Homan PJ Agrawal H Misharin AV Abdala-Valencia H Haines GK 3rd. Bim suppresses the development of SLE by limiting myeloid inflammatory responses. J Exp Med (2017) 214:3753–73. doi: 10.1084/jem.20170479 PMC5716039 29114065

[17] Padgett LE Burg AR Lei W Tse HM. Loss of NADPH oxidase-derived superoxide skews macrophage phenotypes to delay type 1 diabetes. Diabetes (2015) 64:937–46. doi: 10.2337/db14-0929 PMC4338593 25288672

[18] Wang B He W Li Q Jia X Yao Q Song R. U-Shaped relationship between iodine status and thyroid autoimmunity risk in adults. Eur J Endocrinol (2019) 181:255–66. doi: 10.1530/EJE-19-0212 31252413

[19] Hu S Rayman MP. Multiple nutritional factors and the risk of hashimoto’s thyroiditis. Thyroid (2017) 27:597–610. doi: 10.1089/thy.2016.0635 28290237

[20] Duntas LH. Environmental factors and autoimmune thyroiditis. Nat Clin Pract Endocrinol Metab (2008) 4:454–60. doi: 10.1038/ncpendmet0896 18607401

[21] Bilal MY Dambaeva S Kwak-Kim J Gilman-Sachs A Beaman KD. A role for iodide and thyroglobulin in modulating the function of human immune cells. Front Immunol (2017) 8:1573. doi: 10.3389/fimmu.2017.01573 29187856 PMC5694785

[22] Tang CY Mauro C. Similarities in the metabolic reprogramming of immune system and endothelium. Front Immunol (2017) 8:837. doi: 10.3389/fimmu.2017.00837 28785263 PMC5519526

[23] Xu T Stewart KM Wang X Liu K Xie M Ryu JK. Metabolic control of T(H)17 and induced t(reg) cell balance by an epigenetic mechanism. Nature (2017) 548:228–33. doi: 10.1038/nature23475 PMC6701955 28783731

[24] Binger KJ Côrte-Real BF Kleinewietfeld M. Immunometabolic regulation of interleukin-17-Producing T helper cells: Uncoupling new targets for autoimmunity. Front Immunol (2017) 8:311. doi: 10.3389/fimmu.2017.00311 28377767 PMC5359241

[25] Shen Y Wen Z Li Y Matteson EL Hong J Goronzy JJ. Metabolic control of the scaffold protein TKS5 in tissue-invasive, proinflammatory T cells. Nat Immunol (2017) 18:1025–34. doi: 10.1038/ni.3808 PMC5568495 28737753

[26] Mak TW Grusdat M Duncan GS Dostert C Nonnenmacher Y Cox M. Glutathione primes T cell metabolism for inflammation. Immunity (2017) 46:675–89. doi: 10.1016/j.immuni.2017.03.019 28423341

[27] Ma EH Bantug G Griss T Condotta S Johnson RM Samborska B. Serine is an essential metabolite for effector T cell expansion. Cell Metab (2017) 25:345–57. doi: 10.1016/j.cmet.2016.12.011 28111214

[28] O’Neill LAJ Artyomov MN. Itaconate: the poster child of metabolic reprogramming in macrophage function. Nat Rev Immunol (2019) 19:273–81. doi: 10.1038/s41577-019-0128-5 30705422

[29] Wang F Zhang S Jeon R Vuckovic I Jiang X Lerman A. Interferon gamma induces reversible metabolic reprogramming of M1 macrophages to sustain cell viability and pro-inflammatory activity. EBioMedicine (2018) 30:303–16. doi: 10.1016/j.ebiom.2018.02.009 PMC5953001 29463472

[30] Yoon BR Oh YJ Kang SW Lee EB Lee WW. Role of SLC7A5 in metabolic reprogramming of human Monocyte/Macrophage immune responses. Front Immunol (2018) 9:53. doi: 10.3389/fimmu.2018.00053 29422900 PMC5788887

[31] Kolde R Laur S Adler P Vilo J. Robust rank aggregation for gene list integration and meta-analysis. Bioinformatics (2012) 28:573–80. doi: 10.1093/bioinformatics/btr709 PMC3278763 22247279

[32] Subramanian A Tamayo P Mootha VK Mukherjee S Ebert BL Gillette MA. Gene set enrichment analysis: A knowledge-based approach for interpreting genome-wide expression profiles. Proc Natl Acad Sci U.S.A. (2005) 102:15545–50. doi: 10.1073/pnas.0506580102 PMC1239896 16199517

[33] Hung JH Yang TH Hu Z Weng Z DeLisi C. Gene set enrichment analysis: performance evaluation and usage guidelines. Brief Bioinform (2012) 13:281–91. doi: 10.1093/bib/bbr049 PMC3357488 21900207

[34] Jia X Zhai T Qu C Ye J Zhao J Liu X. Metformin reverses hashimoto’s thyroiditis by regulating key immune events. Front Cell Dev Biol (2021) 9:685522. doi: 10.3389/fcell.2021.685522 34124070 PMC8193849

[35] Newman AM Liu CL Green MR Gentles AJ Feng W Xu Y. Robust enumeration of cell subsets from tissue expression profiles. Nat Methods (2015) 12:453–7. doi: 10.1038/nmeth.3337 PMC4739640 25822800

[36] Langfelder P Horvath S. WGCNA: An r package for weighted correlation network analysis. BMC Bioinf (2008) 9:559. doi: 10.1186/1471-2105-9-559 PMC2631488 19114008

[37] Arora H Wilcox SM Johnson LA Munro L Eyford BA Pfeifer CG. The ATP-binding cassette gene ABCF1 functions as an E2 ubiquitin-conjugating enzyme controlling macrophage polarization to dampen lethal septic shock. Immunity (2019) 50:418–31.e6. doi: 10.1016/j.immuni.2019.01.014 30770245

[38] Raimondo TM Mooney DJ. Functional muscle recovery with nanoparticle-directed M2 macrophage polarization in mice. Proc Natl Acad Sci USA (2018) 115:10648–53. doi: 10.1073/pnas.1806908115 PMC6196479 30275293

[39] Ruytinx P Proost P Van Damme J Struyf S. Chemokine-induced macrophage polarization in inflammatory conditions. Front Immunol (2018) 9:1930. doi: 10.3389/fimmu.2018.01930 30245686 PMC6137099

[40] Jha AK Huang SC Sergushichev A Lampropoulou V Ivanova Y Loginicheva E. Network integration of parallel metabolic and transcriptional data reveals metabolic modules that regulate macrophage polarization. Immunity (2015) 42:419–30. doi: 10.1016/j.immuni.2015.02.005 25786174

[41] Stienstra R Netea-Maier RT Riksen NP Joosten LAB Netea MG. Specific and complex reprogramming of cellular metabolism in myeloid cells during innate immune responses. Cell Metab (2017) 26:142–56. doi: 10.1016/j.cmet.2017.06.001 28683282

[42] Jordão MJC Sankowski R Brendecke SM Sagar Locatelli G Tai YH. Single-cell profiling identifies myeloid cell subsets with distinct fates during neuroinflammation. Science (2019) 363:eaat7554. doi: 10.1126/science.aat7554 30679343

[43] Singh P Dejager L Amand M Theatre E Vandereyken M Zurashvili T. DUSP3 genetic deletion confers M2-like macrophage-dependent tolerance to septic shock. J Immunol (2015) 194:4951–62. doi: 10.4049/jimmunol.1402431 PMC4417402 25876765

[44] Bazzan E Turato G Tinè M Radu CM Balestro E Rigobello C. Dual polarization of human alveolar macrophages progressively increases with smoking and COPD severity. Respir Res (2017) 18:40. doi: 10.1186/s12931-017-0522-0 28231829 PMC5324331

[45] Chang HH Miaw SC Tseng W Sun YW Liu CC Tsao HW. PTPN22 modulates macrophage polarization and susceptibility to dextran sulfate sodium-induced colitis. J Immunol (2013) 191:2134–43. doi: 10.4049/jimmunol.1203363 23913970

[46] Li M Beauchemin H Popovic N Peterson A d’Hennezel E Piccirillo CA. The common, autoimmunity-predisposing 620Arg > trp variant of PTPN22 modulates macrophage function and morphology. J Autoimmun (2017) 79:74–83. doi: 10.1016/j.jaut.2017.01.009 28237724

[47] Zhang WC Zheng XJ Du LJ Sun JY Shen ZX Shi C. High salt primes a specific activation state of macrophages, M(Na). Cell Res (2015) 25:893–910. doi: 10.1038/cr.2015.87 26206316 PMC4528058

[48] Binger KJ Gebhardt M Heinig M Rintisch C Schroeder A Neuhofer W. High salt reduces the activation of IL-4- and IL-13-stimulated macrophages. J Clin Invest (2015) 125:4223–38. doi: 10.1172/JCI80919 PMC4639967 26485286

[49] Dionne S Duchatelier CF Seidman EG. The influence of vitamin d on M1 and M2 macrophages in patients with crohn’s disease. Innate Immun (2017) 23:557–65. doi: 10.1177/1753425917721965 28770666

[50] Zhu X Zhu Y Li C Yu J Ren D Qiu S. 1,25−Dihydroxyvitamin d regulates macrophage polarization and ameliorates experimental inflammatory bowel disease by suppressing miR-125b. Int Immunopharmacol (2019) 67:106–18. doi: 10.1016/j.intimp.2018.12.015 30540970

[51] O’Neill LA Kishton RJ Rathmell J. A guide to immunometabolism for immunologists. Nat Rev Immunol (2016) 16:553–65. doi: 10.1038/nri.2016.70 PMC5001910 27396447

[52] Koelwyn GJ Corr EM Erbay E Moore KJ. Regulation of macrophage immunometabolism in atherosclerosis. Nat Immunol (2018) 19:526–37. doi: 10.1038/s41590-018-0113-3 PMC6314674 29777212

[53] Wang F Wang K Xu W Zhao S Ye D Wang Y. SIRT5 desuccinylates and activates pyruvate kinase M2 to block macrophage IL-1β production and to prevent DSS-induced colitis in mice. Cell Rep (2017) 19:2331–44. doi: 10.1016/j.celrep.2017.05.065 28614718

[54] Xie M Yu Y Kang R Zhu S Yang L Zeng L. PKM2-dependent glycolysis promotes NLRP3 and AIM2 inflammasome activation. Nat Commun (2016) 7:13280. doi: 10.1038/ncomms13280 27779186 PMC5093342

[55] Palsson-McDermott EM Curtis AM Goel G Lauterbach MA Sheedy FJ Gleeson LE. Pyruvate kinase M2 regulates hif-1α activity and IL-1β induction and is a critical determinant of the warburg effect in LPS-activated macrophages. Cell Metab (2015) 21:65–80. doi: 10.1016/j.cmet.2014.12.005 25565206 PMC5198835

[56] Finucane OM Sugrue J Rubio-Araiz A Guillot-Sestier MV Lynch MA. The NLRP3 inflammasome modulates glycolysis by increasing PFKFB3 in an IL-1β-dependent manner in macrophages. Sci Rep (2019) 9:4034. doi: 10.1038/s41598-019-40619-1 30858427 PMC6411754

[57] Jiang H Shi H Sun M Wang Y Meng Q Guo P. PFKFB3-driven macrophage glycolytic metabolism is a crucial component of innate antiviral defense. J Immunol (2016) 197:2880–90. doi: 10.4049/jimmunol.1600474 27566823

[58] Moon JS Hisata S Park MA DeNicola GM Ryter SW Nakahira K. mTORC1-induced HK1-dependent glycolysis regulates NLRP3 inflammasome activation. Cell Rep (2015) 12:102–15. doi: 10.1016/j.celrep.2015.05.046 PMC4858438 26119735

[59] Bustamante MF Oliveira PG Garcia-Carbonell R Croft AP Smith JM Serrano RL. Hexokinase 2 as a novel selective metabolic target for rheumatoid arthritis. Ann Rheum Dis (2018) 77:1636–43. doi: 10.1136/annrheumdis-2018-213103 PMC6328432 30061164

[60] Perrin-Cocon L Aublin-Gex A Diaz O Ramière C Peri F André P. Toll-like receptor 4-induced glycolytic burst in human monocyte-derived dendritic cells results from p38-dependent stabilization of HIF-1α and increased hexokinase II expression. J Immunol (2018) 201:1510–21. doi: 10.4049/jimmunol.1701522 30037846

[61] Blagih J Jones RG. Polarizing macrophages through reprogramming of glucose metabolism. Cell Metab (2012) 15:793–5. doi: 10.1016/j.cmet.2012.05.008 22682218

[62] Zhang J Wang S Jiang B Huang L Ji Z Li X. C-src phosphorylation and activation of hexokinase promotes tumorigenesis and metastasis. Nat Commun (2017) 8:13732. doi: 10.1038/ncomms13732 28054552 PMC5227066

[63] Woldetsadik AD Vogel MC Rabeh WM Magzoub M. Hexokinase II-derived cell-penetrating peptide targets mitochondria and triggers apoptosis in cancer cells. FASEB J (2017) 31:2168–84. doi: 10.1096/fj.201601173R PMC5388548 28183803

[64] Gao Y Yang Y Yuan F Huang J Xu W Mao B. TNFα-YAP/p65-HK2 axis mediates breast cancer cell migration. Oncogenesis (2017) 6:e383. doi: 10.1038/oncsis.2017.83 28945218 PMC5623908

