# Supplementary figures and images for: High iodine promotes autoimmune thyroid disease by activating hexokinase 3 and inducing polarization of macrophages towards M1
Source: Front Immunol. 2022 Oct 17;13:1009932. doi: 10.3389/fimmu.2022.1009932 (PMC9618622; doi:10.3389/fimmu.2022.1009932)

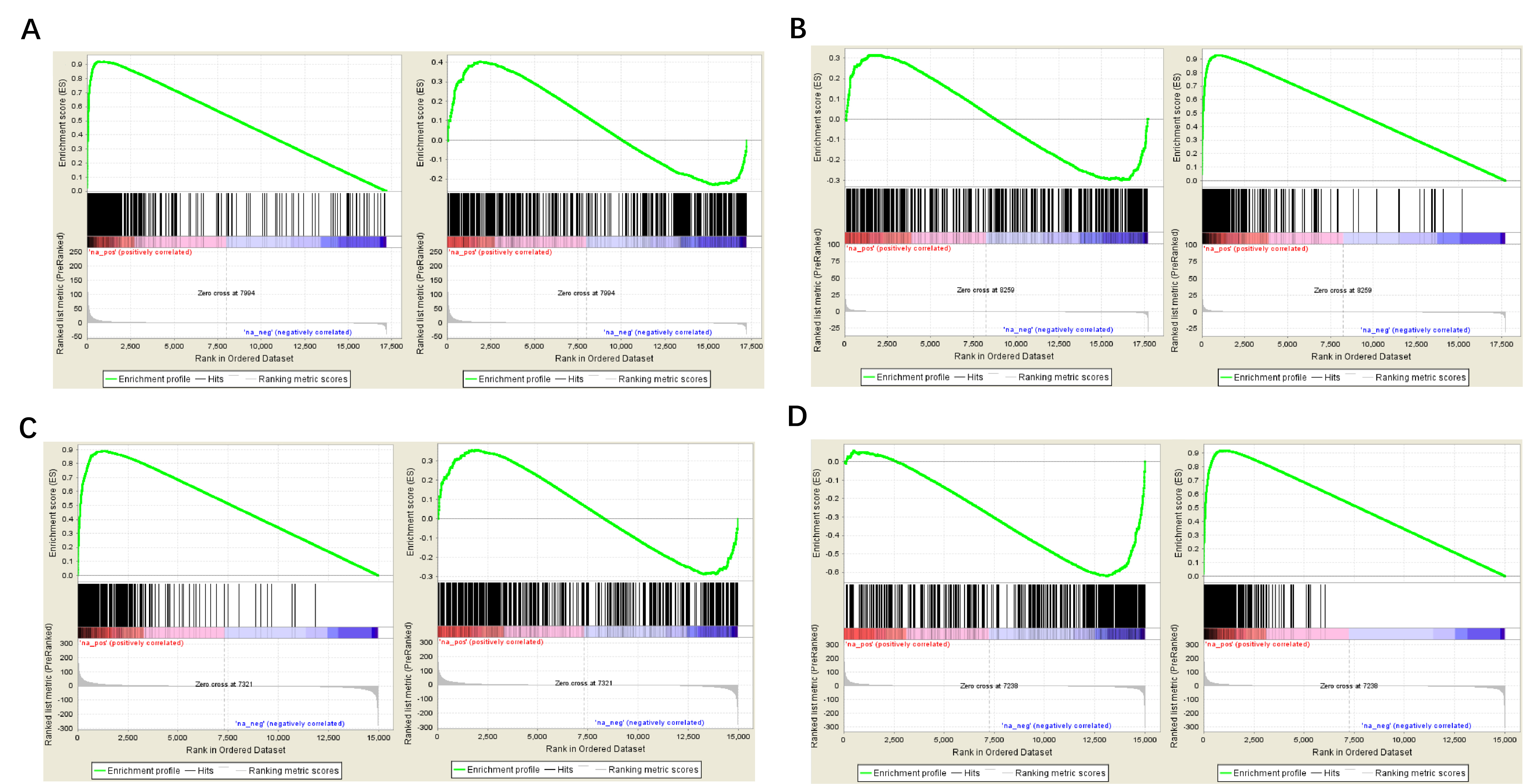

Supplement: Supplementary Figure 1 — GSEA validation of human and mouse macrophage polarization gene sets. (A) Enrichment plots of human macrophage polarization gene sets in the GSEA analysis of human M1 polarization induced by IFN-γ in GSE82227 (From left to right: M1 and M2; M1: ES=0.92 (P<0.001, FDR q <0.001; M2: ES=0.40, P=0.91, FDR q=0.91); (B) Enrichment plots of human macrophage polarization gene sets in the GSEA analysis of human M2 polarization induced by IL-4 in GSE123603(From left to right: M1 and M2; M1: ES=0.31, P=0.67, FDR q=0.68; M2: ES=0.93, P<0.001, FDR q <0.001); (C) Enrichment plots of mice macrophage polarization gene sets in the GSEA analysis of mice M1 polarization induced by LPS in GSE123180(From left to right: M1 and M2; M1:ES=0.89,P<0.001, FDR q <0.001; M2: ES=0.35, P=0.36, FDR q=0.37); (D) Enrichment plots of mice macrophage polarization gene sets in the GSEA analysis of mice M2 polarization induced by IL-4 in GSE123180 (From left to right: M1 and M2; M1: ES= -0.62, P<0.001, FDR q <0.001;M2: ES=0.92,P<0.001, FDR q <0.001). [file Image_1.jpeg]

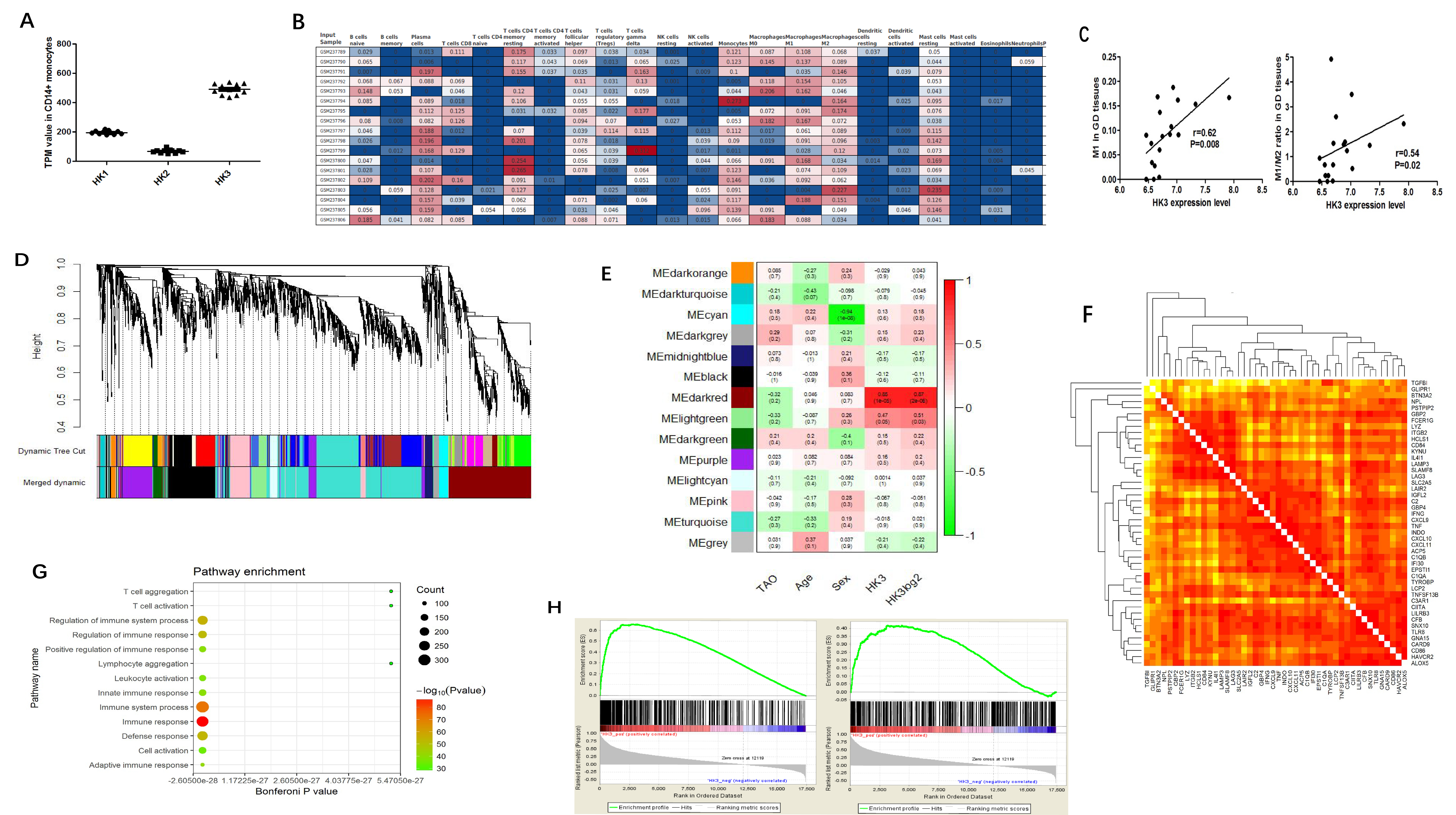

Supplement: Supplementary Figure 2 — Systems biology revealed the role of HK3 in the pathogenesis of GD. (A) HK3 expression abundance in CD14+ monocyte-macrophages; (B) The proportion of major immune cells in 18 thyroid tissue in the GSE9340 dataset; (C) The relationship between HK3 and the proportion of M1 macrophages and M1/M2 ratio; (D) HK3-related co-expression gene modules by WGCNA analysis; (E) Darkred module was most correlated with HK3; (F) Heatmap of key genes of Darkred module; (G) GO analysis; (H) GSEA analysis. [file Image_2.jpeg]
