# Supplementary material for: High iodine promotes autoimmune thyroid disease by activating hexokinase 3 and inducing polarization of macrophages towards M1
Source: Front Immunol. 2022 Oct 17;13:1009932. doi: 10.3389/fimmu.2022.1009932 (PMC9618622; doi:10.3389/fimmu.2022.1009932)
Supplement: Supplementary file 3 [file DataSheet_1.docx]

**Table S1 Primer sequences for qRT-PCR**

| **Genes** | **Primer sequences** |
| --- | --- |
| Mouse | |
| Gapdh | Forward: AGGTCGGTGTGAACGGATTTG |
|  | Reverse: TGTAGACCATGTAGTTGAGGTCA |
| Il6 | Forward: TAGTCCTTCCTACCCCAATTTCC |
|  | Reverse: TTGGTCCTTAGCCACTCCTTC |
| Il1b | Forward: GCAACTGTTCCTGAACTCAACT |
|  | Reverse: ATCTTTTGGGGTCCGTCAACT |
| Tnf | Forward: ATGTCTCAGCCTCTTCTCATTC |
|  | Reverse: GCTTGTCACTCGAATTTTGAGA |
| Il10 | Forward: GCTCTTACTGACTGGCATGAG |
|  | Reverse: CGCAGCTCTAGGAGCATGTG |
| Hk1 | Forward: CGGAATGGGGAGCCTTTGG |
|  | Reverse: GCCTTCCTTATCCGTTTCAATGG |
| Hk2 | Forward: TGATCGCCTGCTTATTCACGG |
|  | Reverse: AACCGCCTAGAAATCTCCAGA |
| Hk3 | Forward: CAGGGGACCTACAGGATTGAT |
|  | Reverse: GAGCATCTTCGTCATAGAAGGAG |
| Human | |
| ACTB | Forward: CATTGCCGACAGGATGCAG |
|  | Reverse: CTCGTCATACTCCTGCTTGCTG |
| IL1B | Forward: GCCAGTGAAATGATGGCTTATT |
|  | Reverse: AGGAGCACTTCATCTGTTTAGG |
| IL6 | Forward: CACTGGTCTTTTGGAGTTTGAG |
|  | Reverse: GGACTTTTGTACTCATCTGCAC |
| HK1 | Forward: GGACTGGACCGTCTGAATGT |
|  | Reverse: ACAGTTCCTTCACCGTCTGG |
| HK2 | Forward: CAAAGTGACAGTGGGTGTGG |
|  | Reverse: GCCAGGTCCTTCACTGTCTC |
| HK3 | Forward: CTCCATTGGGTCTTCAGGGT |
|  | Reverse: CACGAAGTCTCCTTGCTCAGT |

**Table S2 402 key metabolic genes analyzed by RRA**

| **Metabolic pathways** | **Related genes** |
| --- | --- |
| Glucose transporters | SLC2A1, SLC2A2, SLC2A3, SLC2A4, SLC2A5, SLC2A6, SLC2A7, SLC2A8, SLC2A9, SLC2A10, SLC2A11, SLC2A12, SLC2A14 |
| Glycolysis | ADPGK, ALDOA, ALDOB, ALDOC, BPGM, ENO1, ENO2, ENO3, GALM, GAPDH, GAPDHS, GCK, GPI, HK1, HK2, HK3, HKDC1, PFKFB1, PFKFB2, PFKFB3, PFKFB4, PFKL, PFKM, PFKP, PGAM1, PGAM2, PGAM4, PGK1, PGK2, PKL, PKLR, PKM1, PKM2, PKR, TPI1 |
| Lactate production and transporters | LDHA, LDHB, LDHC, LDHAL6A, LDHAL6B, UEVLD, SLC16A1, SLC16A2, SLC16A3, SLC16A4, SLC16A5, SLC16A6, SLC16A7, SLC16A8, SLC16A9, SLC16A10, SLC16A11, SLC16A12 |
| Gluconeogenesis | BCAT1, BCAT2, FBP1, FBP2, G6PC, G6PC2, G6PC3, GPT, GPT2, LDHD, PC, PCK1, PCK2 |
| Glycogen metabolism | AGL, GBE1, GSK3A, GSK3B, GYS1, GYS2, PGM1, PGM2, PGM3, PHKA1, PHKB, PHKG1, PHKG2, PYGL, PYGM, UGP2 |
| Hexosamine metabolism | GFPT1, GFPT2, GNPNAT1, PGM3, UAP1, UAP1L1 |
| Pentose phosphate pathway | G6PD, H6PD, PGD, PGLS, PRPS1, PRPS1L1, PRPS2, RBKS, RPE, RPEL1, RPIA, TALDO1, TKT, TKTL1, TKTL2 |
| Glycerol/fatty acid/cholesterol synthesis | ACACA, ACACB, ACAT1, ACAT2, ACLY, ACSBG1, ACSBG2, ACSL1, ACSL3, ACSL4, ACSL5, ACSL6, ACSM1, ACSM2A, ACSM2B, ACSM3, ACSM4, ACSM5, FADS1, FADS2, FASN, GPD1, GPD1L, HMGCR, HMGCS1, HMGCS2, MLYCD, SCD, SCD5, SLC25A1, SLC27A2 |
| Serine/ glycine/ one-carbon metabolism | AHCY, AHCYL1, AHCYL2, AMT, BHMT, DHFR, DHFRL1, DLD, DNMT1, DNMT3A, DNMT3B, DNMT3L, GCSH, GLDC, MAT1A, MAT2A, MAT2B, MTHFD1, MTHFD1L, MTHFD2, MTHFD2L, MTHFR, MTR, PHGDH, PSAT1, PSPH, SHMT1, SHMT2 |
| TCA cycle | ACO1, ACO2, CS, D2HGDH, DHTKD1, DLAT, DLD, DLST, FH, IDH1, IDH2, IDH3A, IDH3B, IDH3G, L2HGDH, MDH1, MDH1B, MDH2, OGDH, OGDHL, PDHA1, PDHA2, PDHB, PDHX, PDK1, PDK2, PDK3, PDK4, PDP1, PDP2, PDPR, SDHA, SDHAF1, SDHAF2, SDHAF3, SDHAF4, SDHB, SDHC, SDHD, SUCLA2, SUCLG1, SUCLG2, UEVLD |
| Glutamine transporters and glutaminolysis | GLS2, GLS, GLUD1, GLUD2, GOT1, GOT2, SLC1A1, SLC1A2, SLC1A3, SLC1A4, SLC1A5, SLC1A6, SLC38A1, SLC38A3, SLC38A5, SLC38A7 |
| Redox balance | CBS, CTH, G6PD, GCLC, GCLM, GSR, GSS, IDH1, IDH2, ME1, ME2, ME3, MTHFD1, NNT, PGD, SLC7A11 |
| GSH synthesis | CBS, CTH, GCLC, GCLM, GSR, GSS, SLC7A11 |
| Fatty acid oxidation | AADAC, ABHD12, ABHD6, ACAA1, ACAA2, ACAD10, ACAD11, ACAD8, ACAD9, ACADL, ACADM, ACADS, ACADSB, ACADVL, ALDH1B1, ALDH2, ALDH3A2, ALDH7A1, ALDH9A1, CEL, CPT1A, CPT1B, CPT1C, CPT2, ECH1, ECHS1, ECI1, ECI2, EHHADH, ETFA, ETFB, HADH, HADHA, HADHB, HSD17B10, HSD17B4, LIPC, LIPE, LIPF, LIPG, MGLL, PAFAH1B1, PAFAH1B2, PAFAH1B3, PNLIP, PNLIPRP1, PNLIPRP2, PNLIPRP3, PNPLA2, PNPLA3, SCP2 |
| Acetate metabolism | ACOT12, ACSS1, ACSS2, ACSS3 |
| Nucleotide metabolism | ADA, ADCY1, ADCY10, ADCY2, ADCY3, ADCY4, ADCY5, ADCY6, ADCY7, ADCY9, ADSL, ADSS, ADSSL1, AK1, AK2, AK3, AK4, AK5, AK6, AK7, AK8, AK9, AMPD1, AMPD2, AMPD3, APRT, ATIC, CAD, CANT1, CDA, CECR1, CMPK1, CMPK2, CTPS1, DCK, DCTD, DHODH, DTYMK, DUT, GART, GDA, GMPS, GUCA1A, GUCA1B, GUCA1C, GUCA2A, GUCA2B, GUCD1, GUCY1A2, GUCY1A3, GUCY1B3, GUCY2C, GUCY2D, GUCY2F, GUK1, HPRT1, IMPDH1, IMPDH2, NME1, NME2, NME3, NME4, NME6, NME7, NT5C2, PAICS, PDE10A, PDE4D, PFAS, PNP, PPAT, RRM1, RRM2, RRM2B, TK1, TK2, TYMP, TYMS, UCKL1, UMPS, UPP1, UPP2, XDH |
| Arginine metabolism | AOC1, ACY1, AGMAT, ALDH18A1, ALDH4A1, ALDH9A1, AMD1, ARG1, ARG2, ASL, ASS1, CKB, CPS1, GAMT, GATM, GLUD1, GOT2, MAOB, NAGS, NOS2, OAT, OTC, SAT1, SRM |

**Table S3 Datasets on polarization of human M1 macrophages**

| **Series** | **Cells** | **Group (replicates)** | **Enrichment type** | **Platforms** |
| --- | --- | --- | --- | --- |
| GSE61298 | Human MDMs | LPS+IFN-γ: 3; Control: 3 | Array | GPL15207 |
| GSE99056 | Human MDMs | LPS: 3; Control: 3 | Array | GPL13497 |
| GSE86298 | Human MDMs | IFN-γ: 4; Control: 4 | Array | GPL20171 |
| GSE83957 | Human MDMs | IFN-γ: 4; Control: 4 | Array | GPL10558 |
| GSE57614 | Human MDMs | LPS+IFN-γ: 3; Control: 3 | Array | GPL6480 |
| GSE36537 | Human MDMs | FN-γ: 3; Control: 3 | Array | GPL6480 |
| GSE36933 | Human MDMs | LPS: 4; Control: 4 | Array | GPL6244 |
| GSE5099 | Human MDMs | LPS+IFN-γ: 3; Control: 3 | Array | GPL97 |
| GSE18686 | Human MDMs | IFN-γ: 6; LPS: 6; LPS+ IFN-γ: 6; Control: 5; | Array | GPL6947 |
| GSE90010 | Human MDMs | LPS: 4; Control: 4 | Array | GPL17692 |
| GSE100382 | Human MDMs | LPS: 3; IFN-γ: 3; LPS+IFN-γ: 3; Control: 3 | Sequencing | GPL11154 |
| GSE76802 | Human MDMs | LPS: 3; Control: 3 | Array | GPL10558 |
| GSE79077 | Human MDMs | IFN-γ: 3; Control: 3 | Array | GPL17077 |
| GSE76561 | Human MDMs | LPS: 3; Control: 3 | Array | GPL15034 |
| GSE43596 | Human MDMs | LPS: 6; Control: 6 | Array | GPL570 |
| GSE32282 | Human MDMs | LPS: 3; Control: 3 | Array | GPL6480 |
| GSE49709 | Human MDMs | LPS: 3; Control: 3 | Array | GPL10558 |
| GSE41295 | Human MDMs | LPS: 4; Control: 4 | Array | GPL5175 |
| GSE30177 | Human MDMs | LPS: 3; Control: 3 | Array | GPL10739 |
| GSE19482 | Human MDMs | LPS: 4; Control: 4 | Array | GPL9801 |
| GSE1925 | Human MDMs | IFN-γ: 3; Control: 3 | Array | GPL8300 |
| GSE97744 | Human MDMs | LPS+IFN-γ: 2; Control: 2 | Sequencing | GPL16791 |
| GSE98368 | Human MDMs | IFN-γ: 2; Control: 2 | Sequencing | GPL11154 |
| GSE83381 | Human MDMs | LPS: 2; Control: 2 | Sequencing | GPL11154 |

MDMs, monocyte-derived macrophages

**Table S4 Datasets on polarization of human M2 macrophages**

| **Series** | **Cells** | **Group(replicates)** | **Enrichment type** | **Platforms** |
| --- | --- | --- | --- | --- |
| GSE61298 | Human MDMs | IL-4: 3; Control: 3 | Array | GPL15207 |
| GSE86298 | Human MDMs | IL-4: 4; Control: 4 | Array | GPL20171 |
| GSE83957 | Human MDMs | IL-4: 4; Control: 4 | Array | GPL10558 |
| GSE55029 | Human MDMs | IL-4: 3; Control: 3 | Array | GPL10558 |
| GSE57614 | Human MDMs | IL-4: 3; Control: 3 | Array | GPL6480 |
| GSE36537 | Human MDMs | IL-4: 3; Control: 3 | Array | GPL6480 |
| GSE5099 | Human MDMs | IL-4: 3; Control: 3 | Array | GPL97 |
| GSE35433 | Human MDMs | IL-4: 3; Control: 3 | Array | GPL6947 |
| GSE32164 | Human MDMs | IL-4: 3; Control: 3 | Array | GPL6244 |
| GSE18686 | Human MDMs | IL-4: 6; Control: 5; | Array | GPL6947 |
| GSE16385 | Human MDMs | IL-4: 3; Control: 3 | Array | GPL570 |
| GSE97744 | Human MDMs | IL-4(IL-13): 2; Control: 2 | Sequencing | GPL16791 |
| GSE100889 | Human MDMs | IL-4: 2; Control: 2 | Sequencing | GPL16791 |

MDMs, monocyte-derived macrophages

**Table S5 Datasets on polarization of mouse M1 macrophages**

| **Series** | **Cells** | **Group(replicates)** | **Enrichment type** | **Platforms** |
| --- | --- | --- | --- | --- |
| GSE113009 | Mice BMDMs | LPS: 2; Control: 2 | Sequencing | GPL13112 |
| GSE123289 | Mice BMDMs | LPS+IFN-γ: 4; Control: 4 | Array | GPL21163 |
| GSE125036 | Mice BMDMs | LPS: 3; Control: 3 | Sequencing | GPL21626 |
| GSE124774 | Mice BMDMs | LPS+IFN-γ: 6; Control: 6 | Sequencing | GPL21103 |
| GSE109997 | Mice BMDMs | LPS: 3; Control: 3 | Sequencing | GPL19057 |
| GSE122070 | Mice BMDMs | LPS: 2; Control: 2 | Sequencing | GPL21103 |
| GSE103958 | Mice BMDMs/RAW 264.7 | LPS+IFN-γ: 3; Control: 3; LPS(RAW 264.7): 2; Control(RAW 264.7): 2 | Sequencing | GPL17021 |
| GSE106700 | Mice BMDMs | LPS: 3; Control: 3 | Sequencing | GPL17021 |
| GSE98840 | Mice BMDMs | LPS: 8; Control: 8 | Array | GPL21382 |
| GSE84517 | Mice BMDMs | IFN-γ: 4; Control: 4 | Sequencing | GPL13112 |
| GSE80185 | Mice BMDMs | IFN-γ: 6; Control: 6 | Array | GPL19462 |
| GSE72518 | Mice BMDMs | LPS+IFN-γ: 3; Control: 5 | Array | GPL1261 |
| GSE68167 | Mice BMDMs | LPS: 3; Control: 3 | Array | GPL6887 |
| GSE73310 | Mice peritoneal macrophages | LPS+IFN-γ: 4; Control: 4 | Array | GPL10787 |
| GSE69607 | Mice BMDMs | LPS+IFN-γ: 3; Control: 3 | Array | GPL1261 |
| GSE53321 | Mice BMDMs | LPS+IFN-γ: 3; Control: 3 | Array | GPL1261 |
| GSE53053 | Mice BMDMs | LPS+IFN-γ: 3; Control: 2 | Sequencing | GPL17021 |
| GSE32690 | Mice BMDMs | IFN-γ: 3; LPS+IFN-γ: 3; Control: 3 | Array | GPL7202 |
| GSE41833 | Mice BMDMs | LPS: 3; Control: 4 | Array | GPL11533 |
| GSE81291 | Mice BMDMs | LPS: 3; Control: 3 | Array | GPL10787 |
| GSE98506 | Mice BMDMs | LPS: 3; Control: 3 | Sequencing | GPL17021 |
| GSE100887 | Mice RAW 264.7 | LPS: 3; Control: 3 | Sequencing | GPL13112 |
| GSE86588 | Mice RAW 264.7 | LPS: 3; Control: 3 | Array | GPL10787 |
| GSE100059 | Mice BMDMs | LPS: 3; Control: 3 | Array | GPL23585 |
| GSE89559 | Mice BMDMs | LPS+IFN-γ: 3; Control: 3 | Array | GPL6887 |
| GSE70155 | Mice BMDMs | LPS: 3; Control: 3 | Array | GPL6887 |
| GSE78849 | Mice BMDMs | LPS: 2; Control: 3 | Sequencing | GPL16417 |
| GSE87369 | Mice RAW 264.7 | LPS: 4; Control: 4 | Array | GPL11533 |
| GSE86020 | Mice peritoneal macrophages | LPS: 3; Control: 3 | Array | GPL21810 |
| GSE58318 | Mice BMDMs | LPS+IFN-γ: 3; Control: 3 | Sequencing | GPL13112 |
| GSE82043 | Mice BMDMs | LPS: 3; Control: 3 | Sequencing | GPL17021 |
| GSE108581 | Mice BMDMs | LPS: 3; LPS+IFN-γ: 3; Control: 3 | Sequencing | GPL17021 |
| GSE97062 | Mice BMDMs | LPS: 6; Control: 6 | Sequencing | GPL23479 |
| GSE82109 | Mice BMDMs | IFN-γ: 3; LPS: 3; LPS+ IFN-γ: 3; Control: 3 | Array | GPL17400 |
| GSE79397 | Mice RAW 264.7 | LPS: 4; Control: 4 | Array | GPL10787 |
| GSE77104 | Mice BMDMs | LPS: 4; Control: 3 | Array | GPL6246 |
| GSE76562 | Mice RAW 264.7 | LPS: 3; Control: 3 | Array | GPL18802 |
| GSE62746 | Mice BMDMs | LPS: 3; Control: 3 | Array | GPL6246 |
| GSE45234 | Mice BMDMs | LPS: 3; Control: 3 | Array | GPL1261 |
| GSE68449 | Mice BMDMs | LPS: 3; Control: 3 | Array | GPL10787 |
| GSE46361 | Mice BMDMs | LPS: 6; Control: 6 | Array | GPL6885 |
| GSE60290 | Mice BMDMs | IFN-γ: 3; LPS: 2; LPS+ IFN-γ: 3; Control: 3 | Array | GPL6885 |
| GSE66782 | Mice peritoneal macrophages | LPS: 3; Control: 3 | Array | GSE66782 |
| GSE58432 | Mice RAW 264.7 | LPS: 3; Control: 3 | Sequencing | GPL18635 |
| GSE50542 | Mice BMDMs | LPS: 4; Control: 4 | Array | GPL13912 |
| GSE54155 | Mice peritoneal macrophages | LPS: 4; Control: 4 | Array | GPL4134 |
| GSE39349 | Mice BMDMs | IFN-γ: 6; Control: 6 | Array | GPL6246 |
| GSE48970 | Mice BMDMs | IFN-γ: 3; Control: 3 | Array | GPL6246 |
| GSE53986 | Mice BMDMs | IFN-γ: 4; LPS: 4; LPS+ IFN-γ: 4; Control: 4 | Array | GPL1261 |
| GSE42190 | Mice peritoneal macrophages | LPS: 4; Control: 3 | Array | GPL6887 |
| GSE47783 | Mice BMDMs | LPS: 3; Control: 3 | Array | GPL13912 |
| GSE48609 | Mice BMDMs | LPS: 3; IFN-γ: 3; Control: 3 | Array | GPL6887 |
| GSE47530 | Mice BMDMs | LPS: 3; Control: 3 | Array | GPL6246 |
| GSE43075 | Mice peritoneal macrophages | LPS: 3; Control: 3 | Array | GPL6246 |
| GSE33162 | Mice BMDMs | LPS: 3; Control: 3 | Array | GPL6246 |
| GSE30971 | Mice BMDMs | LPS: 3; Control: 3 | Array | GPL6246 |
| GSE26343 | Mice BMDMs | LPS: 4; Control: 4 | Array | GPL6246 |
| GSE44294 | Mice BMDMs | LPS: 3; Control: 3 | Array | GPL8321 |
| GSE19490 | Mice BMDMs | LPS: 3; Control: 3 | Array | GPL9802 |
| GSE32359 | Mice BMDMs | LPS: 3; Control: 3 | Array | GPL14615 |
| GSE35825 | Mice BMDMs | IFN-γ: 3; Control: 3 | Array | GPL1261 |
| GSE2002 | Mice RAW 264.7 | LPS: 3; Control: 3 | Array | GPL339 |
| GSE8621 | Mice BMDMs | LPS: 3; Control: 3 | Array | GPL1261 |
| GSE9632 | Mice RAW 264.7 | LPS: 3; Control: 3 | Array | GPL2995 |
| GSE14769 | Mice BMDMs | LPS: 3; Control: 3 | Array | GPL1261 |
| GSE15610 | Mice BMDMs | LPS: 3; Control: 3 | Array | GPL1261 |
| GSE20207 | Mice BMDMs | LPS: 3; Control: 3 | Array | GPL4134 |
| GSE21841 | Mice RAW 264.7 | LPS: 3; Control: 3 | Array | GPL1261 |
| GSE21895 | Mice BMDMs | IFN-γ: 3; LPS: 7; LPS+IFN-γ: 3; Control: 7 | Array | GPL7202 |
| GSE113594 | Mice BMDMs | LPS: 2; Control: 2 | Sequencing | GPL13112 |
| GSE99296 | Mice BMDMs | LPS: 2; Control: 2 | Sequencing | GPL17021 |
| GSE93598 | Mice BMDMs | LPS: 2; Control: 2 | Sequencing | GPL13112 |
| GSE60857 | Mice BMDMs | LPS: 2; Control: 2 | Sequencing | GPL13112 |
| GSE77885 | Mice BMDMs | IFN-γ: 2; Control: 2 | Sequencing | GPL13112 |
| GSE110749 | Mice BMDMs | LPS+IFN-γ: 2; Control: 2 | Sequencing | GPL17021 |
| GSE98810 | Mice BMDMs | LPS: 2; Control: 2 | Sequencing | GPL17021 |
| GSE110243 | Mice BMDMs | LPS: 2; Control: 2 | Sequencing | GPL17021 |
| GSE100216 | Mice BMDMs | LPS: 2; Control: 2 | Sequencing | GPL21103 |
| GSE93735 | Mice BMDMs | LPS: 2; Control: 2 | Sequencing | GPL13112 |
| GSE60930 | Mice RAW 264.7 | LPS: 2; Control: 2 | Sequencing | GPL16331 |
| GSE86922 | Mice BMDMs | LPS: 2; Control: 2 | Sequencing | GPL19057 |

BMDMs, bone marrow-derived macrophage

**Table S6 Datasets on polarization of mouse M2 macrophages**

| **Series** | **Cells** | Group(replicates) | **Enrichment type** | **Platforms** |
| --- | --- | --- | --- | --- |
| GSE123289 | Mice BMDMs | IL-4: 4; Control: 4 | Array | GPL21163 |
| GSE103958 | Mice BMDMs/RAW 264.7 | IL-4: 3; Control: 3 | Sequencing | GPL17021 |
| GSE106704 | Mice BMDMs | IL-4: 4; Control: 4 | Sequencing | GPL17021 |
| GSE106700 | Mice BMDMs | IL-4: 3; Control: 3 | Sequencing | GPL17021 |
| GSE84517 | Mice BMDMs | IL-4: 4; Control: 4 | Sequencing | GPL13112 |
| GSE80185 | Mice BMDMs | IL-4: 6; Control: 6 | Array | GPL19462 |
| GSE72518 | Mice BMDMs | IL4(IL13): 3; Control: 5 | Array | GPL1261 |
| GSE68167 | Mice BMDMs | IL-4: 3; Control: 3 | Array | GPL6887 |
| GSE73309 | Mice peritoneal macrophages | IL-4: 4; Control: 4 | Array | GPL10787 |
| GSE53321 | Mice BMDMs | IL4(IL13): 3; Control: 3 | Array | GPL1261 |
| GSE53053 | Mice BMDMs | IL-4: 3; Control: 2 | Sequencing | GPL17021 |
| GSE32690 | Mice BMDMs | IL-4: 3; Control: 3 | Array | GPL7202 |
| GSE35435 | Mice BMDMs | IL-4: 3; Control: 3 | Array | GPL1261 |
| GSE33608 | Mice BMDMs | IL-4: 3; Control: 4 | Array | GPL4134 |
| GSE70626 | Mice BMDMs | IL-4: 5; Control: 8 | Array | GPL8321 |
| GSE58318 | Mice BMDMs | IL-4: 3; Control: 3 | Sequencing | GPL13112 |
| GSE80160 | Mice peritoneal macrophages | IL-4: 3; Control: 3 | Sequencing | GPL17021 |
| GSE54679 | Mice peritoneal macrophages | IL-4: 4; Control: 3 | Array | GPL13912 |
| GSE25088 | Mice BMDMs | IL-4: 3; Control: 3 | Array | GPL1261 |
| GSE21853 | Mice BMDMs | IL-4: 3; Control: 3 | Array | GPL4134 |
| GSE21895 | Mice BMDMs | IL-4: 4; Control: 7 | Array | GPL7202 |
| GSE113594 | Mice BMDMs | IL-4: 2; Control: 2 | Sequencing | GPL13112 |
| GSE98964 | Mice BMDMs | IL-4: 2; Control: 2 | Sequencing | GPL13112 |
| GSE99296 | Mice BMDMs | IL-4: 2; Control: 2 | Sequencing | GPL17021 |
| GSE98168 | Mice BMDMs | IL-4: 2; Control: 2 | Sequencing | GPL17021 |

BMDMs, bone marrow-derived macrophage

**Table S7 M1 and M2 polarization characteristic gene sets of human macrophages**

| **Gene sets** | **Genes** |
| --- | --- |
| Human M1 | ABL2, ABTB2, ACOT9, ACOX3, ACSL1, ADA, ADAR, ADM, ADORA2A, ADPRHL2, AIM2, ANKRD22, ANTXR2, APOBEC3A, APOBEC3F, APOBEC3G, APOL1, APOL2, APOL3, APOL6, ARHGEF11, ARID5A, ARID5B, ARL5B, ASCL2, ASPHD2, ATF3, ATF5, B4GALT5, BATF2, BAZ1A, BCL2A1, BIRC3, BMP6, BTG1, BTG3, BTN2A1, C15orf48, C18orf8, C1QTNF1, C1R, C21orf91, C5orf15, CASP1, CASP4, CASP5, CASZ1, CBLN3, CCDC50, CCL1, CCL19, CCL2, CCL20, CCL3, CCL4, CCL5, CCL7, CCNA1, CCR7, CCRL2, CD38, CD44, CD48, CD69, CD70, CD80, CDC42EP2, CFB, CFLAR, CKB, CLCF1, CLEC4D, CLEC4E, CLIC4, CMPK2, COQ10B, CSF2, CSF3, CSRNP1, CTRL, CUL1, CXCL1, CXCL10, CXCL11, CXCL2, CXCL9, DAPP1, DCP1A, DDIT4, DDX58, DDX60, DDX60L, DEFB1, DENND5A, DHX58, DNAJA1, DTX3L, DUSP1, DYNLT1, EBI3, EDEM1, EDN1, EHD1, EIF2AK2, ELOVL7, ENDOD1, EPSTI1, EREG, ETV7, EZH2, FAM124A, FAM129A, FAM20A, FAM26F, FAM49A, FAM65B, FAS, FBXL12, FBXO6, FCAMR, FERMT2, FFAR2, FOSL2, FPR2, FUT4, G0S2, GADD45B, GBP1, GBP2, GBP3, GBP4, GBP5, GCH1, GFPT2, GJB2, GK, GMPR, GNE, GPBP1, GPD2, GPR132, GPR84, GRAMD1A, GRAMD3, GRIPAP1, GTF2B, GTPBP1, GUCY1A3, HAPLN3, HCK, HELB, HERC5, HERC6, HES4, HESX1, HIVEP2, HLA-DOB, HLA-F, HS3ST3B1, HSH2D, ICAM1, IDO1, IER3, IFI27, IFI35, IFI44, IFI44L, IFI6, IFIH1, IFIT1, IFIT2, IFIT3, IFIT5, IFITM1, IFITM2, IFITM3, IFRD1, IGF2BP3, IGFBP4, IL10, IL10RA, IL12B, IL15, IL15RA, IL1A, IL1B, IL23A, IL27, IL2RA, IL32, IL4I1, IL6, IL7R, INHBA, IRAK2, IRF1, IRF7, IRF9, IRG1, ISG15, ISG20, ITGB8, JAK2, JUNB, KARS, KCNJ2, KIAA0040, KLF6, KLF9, KYNU, LAG3, LAP3, LCP2, LGALS3BP, LILRA5, LILRB2, LIMK2, LMNB1, LONRF1, LRP10, LRRFIP2, LSS, LY6E, LYN, LYSMD2, MAP1LC3A, MAP3K8, MARCKSL1, MASTL, MCL1, MCOLN2, MESDC1, MICALL1, MICB, MMAA, MMP1, MMP10, MOV10, MSC, MT1A, MT1E, MT1M, MT2A, MTF1, MTHFD2, MUC1, MX1, MX2, MYO10, MYO1G, N4BP1, N4BP2L1, NAMPT, NBN, NCF1, NDP, NEURL3, NEXN, NFE2L3, NFIX, NFKB1, NFKB2, NFKBIA, NFKBIB, NFKBIE, NFKBIZ, NINJ1, NKX3-1, NLRC5, NMI, NOD2, NSUN3, NT5C3, NUB1, OAS1, OAS2, OAS3, OASL, OGFR, OPTN, OSM, OTUD4, P2RX7, PANX1, PARP10, PARP11, PARP12, PARP14, PARP9, PCGF5, PCNX, PDCD1LG2, PDE4B, PDGFRL, PELI1, PFKFB3, PHF11, PHLDA2, PI4K2B, PIM1, PIM2, PLAGL2, PLAUR, PLEKHN1, PLSCR1, PMAIP1, PML, PNPT1, PNRC1, PPA1, PPM1K, PPP1R15A, PPP3CC, PRDM1, PRPF3, PSMA6, PSMB10, PSMB8, PSMB9, PSME1, PSME2, PSTPIP2, PTGER4, PTGES, PTGIR, PTGS2, PTPN1, PTPN2, PTPRJ, PVR, PVRL2, RAB12, RAB24, RAP2C, RAPGEF2, RARRES3, RASGRP1, RBCK1, REC8, RELA, RELB, RHBDF2, RHOH, RILPL2, RIPK1, RIPK2, RNF144B, RNF149, RNF19B, RNF213, RNF31, RSAD2, RTP4, RUFY3, SAMD8, SAMD9, SAMD9L, SAMSN1, SASH1, SAV1, SBNO2, SERPINA1, SERPINB2, SERPINB9, SERPING1, SFT2D2, SGPP2, SIPA1L1, SLAMF7, SLC1A2, SLC22A4, SLC25A28, SLC25A37, SLC2A6, SLC31A2, SLC37A1, SLC38A5, SLC39A8, SLC41A2, SLCO5A1, SLFN5, SNX10, SOCS3, SOD2, SP110, SP140, SPHK1, SQRDL, SSTR2, STAP1, STAT1, STAT2, STAT3, STAT4, STAT5A, STOM, STOML1, STX11, STX17, TAGAP, TANK, TAP1, TAP2, TAPBP, TBC1D22B, TBC1D9, TBK1, TDRD7, TFEC, TICAM1, TLR2, TLR8, TMEM140, TMEM217, TNF, TNFAIP2, TNFAIP3, TNFAIP6, TNFAIP8, TNFRSF9, TNFSF10, TNFSF13B, TNFSF15, TNFSF9, TNIP2, TNIP3, TOR1B, TP53INP2, TRAF3IP2, TRAFD1, TRIM21, TRIM22, TRIM25, TRIM26, TRIM5, TRIM56, TRIP10, TXN, UBE2L6, UPB1, USP18, USP42, VAMP5, VRK2, WARS, WTAP, XAF1, XRN1, YRDC, ZBP1, ZBTB17, ZC3H12A, ZC3HAV1, ZFAND2A, ZFP36, ZNF277, ZNFX1, ZNRF2 |
| Human M2 | ABCG2, ABHD10, ABHD4, ABHD6, ACOT7, ACPL2, ACTA2, ACTN1, ACTR8, ADAM12, ADAM15, ADAMTSL4, ADO, ADPGK, ADPRH, AGPAT4, AIFM1, AIG1, AKAP5, AKIRIN1, ALDH1A2, ALOX15, ALOX15B, AMPD2, ANKMY2, ANPEP, ANXA11, ANXA4, AP1B1, AP2M1, APBB1IP, APEX2, APOL4, AQP3, ARAP2, ARHGAP10, ARHGAP26, ARL4C, ARNTL2, ARRDC4, ARV1, ATP1B1, ATP5G3, ATP6V0A2, ATXN1, AUH, AVPI1, B3GALTL, B3GNT5, BCAR3, BCKDK, BCL2L11, BCL7A, BIRC6, BLVRB, BZW2, C10orf128, C12orf45, C17orf58, C17orf87, C17orf96, C1QB, C1orf162, C3orf18, CACNB4, CAMK1D, CARD9, CBR3, CCDC6, CCL13, CCL17, CCL18, CCL22, CCL26, CCNH, CCRN4L, CD1A, CD1B, CD1C, CD1E, CD200R1, CD209, CD276, CD300A, CD300LB, CD86, CDH1, CDR2, CH25H, CHCHD7, CHD7, CHDH, CHN2, CHST7, CITED2, CLCN5, CLEC10A, CLEC16A, CLEC4A, CLEC4G, CLEC7A, CLIC2, CLNS1A, CMTM7, COPS7B, CR1, CRYZ, CSNK1G1, CTNNAL1, CTNS, CTSC, CYSLTR1, DAAM1, DACT1, DAGLA, DDX21, DENND4C, DHRS11, DIP2B, DIP2C, DNASE1L3, DOCK7, DTNA, ECHDC3, EEA1, EFHD2, EFNA1, EGR2, EHD4, EHF, EMB, EML4, ENHO, EPB41L2, EPN2, EPS15, EPS8, ERI1, ESPNL, ETFB, ETV6, EVL, F13A1, FABP4, FABP5, FAM110B, FAM126A, FAM129B, FAM198A, FAR2, FARP1, FCER1A, FCER2, FCGR2B, FCHO1, FCRLB, FGD2, FGL2, FKBP1A, FLT1, FLVCR2, FN1, FOXQ1, FRMD4A, FST, G6PD, GADD45A, GALNT12, GAS2L3, GAS6, GCN1L1, GDPD5, GFOD1, GGT5, GGTA1P, GPR146, GPR35, GPSM1, GPT2, GSPT1, GSTK1, GSTT1, GTDC1, GTF3C6, GUCA1A, HADHA, HADHB, HDAC2, HEMK1, HES6, HMG20B, HOMER2, HOPX, HS3ST2, HSP90AB1, HSPH1, IDH3A, IFNGR2, IGF2BP2, IL17RB, IL1RAP, IL21R, IL27RA, IL3RA, ILF2, IMP4, ITGA11, ITGAM, ITPRIPL2, JUN, KCNE1, KCNK6, KCTD15, KCTD6, KHSRP, KIAA0100, KLF2, KLF4, KTN1, LARS, LHFP, LIMA1, LIPA, LRRC8B, LRRFIP1, LTV1, LY9, MAF, MAFF, MAN1A1, MAN2A1, MAOA, MAP1A, MAP2K6, MAPK1, MAT2A, MAT2B, MDH1, METRNL, METTL1, METTL7A, MEX3B, MFNG, MFSD3, MGAT1, MID2, MMP12, MOSPD1, MREG, MRPL12, MRPL3, MRPS25, MRPS6, MS4A6A, MYL9, NAGA, NCAPH, NCOR2, NDFIP2, NEK6, NFE2, NFIL3, NFXL1, NIPAL1, NLK, NMD3, NPC1, NR4A3, NUDT16, NUDT16P1, OTUD6B, OVOL1, P2RY12, P2RY14, PALLD, PAM, PAOX, PARM1, PCM1, PCSK5, PCSK7, PDGFB, PDGFC, PDHB, PDP2, PELP1, PER2, PICALM, PIK3C2B, PIK3R1, PITRM1, PKD2, PKIB, PLCB1, PLCB2, POGK, POLD1, PPARG, PPFIBP1, PPFIBP2, PPIC, PPP1R14A, PPP1R16A, PPP1R3B, PPP1R7, PRKD3, PRPS1, PSMG3, PTGFRN, PTGS1, PTPLA, PTPN4, PTPN7, PTPRE, PTPRO, PTRF, QPCT, QPRT, QSOX1, QSOX2, RAB11FIP4, RAB33A, RABEPK, RAD23B, RAI1, RAMP1, RARA, RBM11, RBMS1, RBPJ, RCAN1, REEP5, RHOF, RNF19A, RNF24, ROGDI, RPIA, RRP1B, RRS1, RYBP, RYK, SAMM50, SASH3, SCRN1, SDC4, SELT, SEMA3C, SERPINB6, SERPINE1, SERTAD1, SETMAR, SGK223, SHB, SHMT2, SHPK, SIDT2, SIGLEC10, SLA, SLC22A16, SLC25A25, SLC25A29, SLC25A35, SLC25A48, SLC26A6, SLC27A3, SLC37A3, SLC38A6, SLC45A4, SLC47A1, SLC4A7, SLC7A8, SLCO4C1, SMAD1, SMAD7, SMG7, SMOX, SNAI3, SNX8, SORT1, SPINT1, SPINT2, SPN, SPOCD1, SPRED1, SPRED2, SRGAP1, ST6GAL1, ST7, STAMBPL1, STARD7, STIM2, STYXL1, SUCNR1, SULF2, SUOX, SYNGR2, SYNJ2, SYT17, SYT6, TACSTD2, TAGLN, TBC1D10C, TBC1D8, TGFA, TGFBI, TGM2, THBD, TIAM1, TLE1, TMEM123, TMEM130, TMEM26, TMEM45B, TMEM71, TMTC4, TNFRSF10A, TNFRSF11A, TP53BP2, TRAF5, TRIB1, TRPV2, TSEN2, TSPAN32, TSPAN7, TTC39B, TTC9C, TTLL11, TTYH2, TUBA1A, TUBB6, TXNDC17, UBAP2L, UBE2F, UBE2J1, UBL3, UGP2, UQCRC1, WDR12, WDR36, WDR4, WDR66, WDR74, WDR77, WFS1, WNT5A, WNT5B, XKR3, XPNPEP2, XYLT1, ZC3H8, ZCCHC7, ZDHHC8, ZHX1, ZNF124, ZNF217, ZNF317, ZNF366, ZNF594 |

**Table S8 M1 and M2 polarization characteristic gene sets of mice macrophages**

| **Gene sets** | **Genes** |
| --- | --- |
| Mice M1 | Acsl1, Adamts4, Adar, Adora2a, Adprh, Aff1, Agrn, Agtrap, Aoah, Apobec3, Arg2, Arhgef3, Arid5a, Ass1, Atp10a, Azi2, Batf, Batf2, Bcl2a1c, Bcl3, Birc2, Birc3, Bst1, Bst2, Car13, Casp1, Casp4, Casp7, Cav1, Cbwd1, Ccdc86, Ccdc88b, Ccl22, Ccl3, Ccl4, Ccl5, Ccnd2, Ccrl2, Cd200, Cd38, Cd69, Cd83, Cd86, Cdc42ep2, Cdyl2, Cebpb, Cfb, Cflar, Clec4e, Clic5, Cmpk2, Cnn3, Cp, Creb5, Csf1, Csf3, Ctsc, Cxcl1, Cxcl10, Cxcl11, Cxcl2, Cxcl3, Cxcl9, Daxx, Dcbld2, Dcp1a, Dcp2, Ddx58, Ddx60, Denr, Dgkh, Dhx58, Dst, Dtx2, Dusp1, Dusp16, Dusp2, Ebi3, Ehd1, Eif2ak2, Ell2, Enpp4, Ets2, Etv6, Ext1, Fam26f, Fas, Fcgr4, Fgl2, Flnb, Fnbp1l, Foxp4, Fpr1, Fpr2, Fscn1, Fzd1, Gadd45b, Gbp2, Gbp3, Gbp4, Gbp5, Gbp6, Gbp7, Gca, Gch1, Gem, Ggct, Gja1, Gmeb2, Gpbp1, Gpd2, Gpr132, Gpr18, Gpr84, Gpr85, Gtf2f1, Gtpbp2, H2-T24, Hcar2, Hck, Hdc, Helz2, Herc6, Hif1a, Hivep1, Hivep2, Hk2, Hspa1a, Htra4, Icam1, Ifi203, Ifi204, Ifi205, Ifi35, Ifi44, Ifi47, Ifih1, Ifit1, Ifit2, Ifit3, Ifitm1, Ifnb1, Ifrd1, Igf2bp2, Igtp, Iigp1, Ikbke, Il10, Il12a, Il12b, Il13ra1, Il15, Il15ra, Il18, Il18bp, Il1a, Il1b, Il1f9, Il1rn, Il27, Il2rg, Il4ra, Il6, Inhba, Inpp5b, Irak2, Irak3, Irf1, Irf7, Irg1, Irgm1, Isg15, Isg20, Itga5, Itgal, Jdp2, Junb, Katna1, Kcna3, Kdm6b, Klf6, Klf7, Klra2, Klrk1, Kpna3, Lad1, Lck, Lcn2, Lcp2, Lgals9, Lhx2, Lipg, Lmo4, Lpar1, Lrch1, Lrp11, Lrrc8c, Lta, Lyrm1, Maff, Mafk, Magohb, Malt1, Map3k8, Mapkapk2, Mapkbp1, Marcksl1, Marco, Mcoln2, Mefv, Met, Mfsd7a, Mgat4a, Mitd1, Mlkl, Mmp14, Mmp25, Mov10, Ms4a4c, Ms4a6c, Ms4a6d, Mt2, Mthfr, Mvp, Mx1, Mx2, Mxd1, Myd88, Myo10, N4bp1, Naa25, Nampt, Nfkb1, Nfkb2, Nfkbia, Nfkbib, Nfkbie, Nfkbiz, Nlrc5, Nlrp3, Nmi, Nod1, Nod2, Nos2, Notch1, Nrp2, Nt5c3, Nupr1, Oaf, Oas1a, Oas1b, Oas1g, Oas2, Oas3, Oasl1, Oasl2, Ogfr, Osgin2, Parp11, Parp12, Parp14, Parp8, Parp9, Pcgf5, Pdcd10, Pde4b, Pdpn, Peli1, Pfkfb3, Pgs1, Phlda1, Pilra, Pilrb1, Pla1a, Plagl2, Plaur, Pml, Pnp, Ppa1, Ppm1k, Ppp1r11, Ppp1r15a, Ppp1r15b, Prdx5, Prkrip1, Prkx, Procr, Prpf38a, Psmb8, Psmb9, Psmd10, Pstpip2, Ptges, Ptgs2, Ptpn2, Ptprj, Pvr, Pydc4, Pyhin1, Rab11fip1, Rab12, Rab20, Rab3ip, Rabgef1, Rap2c, Rapgef2, Rbpms, Relb, Rffl, Rhbdf2, Rhou, Rnd1, Rnf135, Rnf14, Rnf24, Rnf31, Rps6ka4, Rrbp1, Rsad2, Rtp4, Saa3, Samd9l, Samhd1, Samsn1, Sav1, Sec24b, Sell, Sema4a, Sepw1, Serpina3f, Serpinb2, Serpine1, Sertad1, Sh3bp4, Shisa3, Skil, Slamf1, Slamf7, Slc23a2, Slc25a22, Slc25a37, Slc28a2, Slc2a6, Slc31a1, Slc31a2, Slc7a11, Slco3a1, Slfn1, Slfn2, Slfn4, Slfn5, Slfn8, Slpi, Smg7, Smpdl3b, Snx10, Socs3, Sod2, Sp100, Sp110, Spata13, Spred1, Srgn, St3gal1, Stat1, Stat2, Stat3, Stk40, Stx11, Stxbp1, Tank, Tap1, Tap2, Tapbp, Tapbpl, Tbk1, Tdrd7, Tgm2, Timp1, Tiparp, Tlk2, Tlr1, Tlr3, Tlr6, Tmem171, Tmem2, Tmem67, Tnf, Tnfaip2, Tnfaip3, Tnfrsf14, Tnfrsf1b, Tnfsf10, Tnfsf15, Tnfsf4, Tnfsf9, Tnip1, Tor1aip2, Tor3a, Tpbg, Tpst1, Traf1, Traf2, Trafd1, Trim13, Trim21, Trim25, Trim26, Trim30a, Trim30d, Triobp, Tspo, Ube2f, Ube2l6, Ugcg, Upp1, Usp12, Usp18, Usp25, Vasp, Vcam1, Vcan, Vps54, Wars, Wdr37, Xaf1, Xkr8, Zbp1, Zc3h12c, Zc3h7a, Zc3hav1, Zcchc2, Zfp281, Zfp36, Zfp513, Zfp800, Zfp811, Znfx1, Zufsp |
| Mice M2 | Abcd2, Abcf3, Abcg1, Ablim1, Aco2, Acp5, Actn1, Actr1b, Acy1, Adam8, Adipoq, Agpat3, Aifm2, Ak2, Ak4, Aldh18a1, Aldh1a2, Alg3, Ampd2, Ang2, Angptl2, Ankrd55, Anxa4, Ap2m1, Apba3, Apex1, Appl1, Aqp3, Aqp9, Arg1, Arhgap10, Arhgap6, Arih1, Arl15, Armc6, Atad3a, Atf3, Atf5, Atic, Atp5b, Atp5d, Atp5g3, Atp6v0a1, Atp6v0d2, Atp6v1b2, Atp6v1h, Auh, B3gnt7, B4galt5, Baiap2, Bak1, Batf3, Bcar3, Bcl2, Bex1, Bhlhe40, Bop1, Brwd1, C1qbp, Car5b, Casp6, Cblb, Ccdc88c, Ccl17, Ccl24, Cct3, Cct8, Cd151, Cd320, Cd44, Cd74, Cdh1, Ceacam19, Chchd10, Chi3l3, Chi3l4, Chic2, Chst7, Ciita, Cisd1, Clcn5, Cldn11, Clec10a, Clec4a3, Clec7a, Clint1, Cln5, Clpp, Clstn1, Cltb, Cops7b, Cox5a, Cox8a, Creld2, Csf2rb, Csf2rb2, Csnk1d, Csnk2b, Ctsz, Cyb5b, Cyc1, Cycs, Dab2ip, Dbt, Ddhd1, Ddx21, Ddx4, Dhcr7, Dhrs9, Dnmt3a, Dok2, Dolpp1, Dusp4, Dut, Ear10, Ear11, Ear3, Eef1e1, Egr2, Eif2b1, Eif2b2, Eif4a1, Eif5a, Elmo2, Eprs, Eps8, Errfi1, Etf1, Etfa, Etfb, Evl, F2r, F7, Fahd1, Fam129b, Fam198b, Fam20c, Fastk, Fchsd2, Fgd6, Flad1, Flrt2, Flt1, Fn1, Fosl2, Foxj3, Fyn, Fzd7, Galnt6, Gar1, Gatm, Gda, Gfm1, Gmppa, Gna15, Gnl3, Gpc1, Gpd1, Grhl1, Grpel1, Grwd1, Gtf3c4, H2-DMa, Hadhb, Hebp2, Hfe, Hhat, Hibadh, Homer1, Hr, Hspa4, Hspa5, Hspa9, Hspd1, Hspe1, Hsph1, Hyou1, Iars, Idh3a, Ier5l, Ifi30, Ifrd2, Igf1, Igsf3, Il1r1, Il1rap, Il1rl1, Il1rl2, Il20rb, Il27ra, Il31ra, Il4i1, Il6st, Inpp5a, Irf4, Itgax, Itgb3, Itpk1, Itsn2, Kcnd3, Kdelr3, Klf4, Klf9, Ksr2, Lancl3, Lap3, Lat, Limk1, Lipn, Lmna, Lpxn, Lrmp, Lrrc32, Ltb4r1, Ltv1, Lyar, Mak16, Man1a, Manbal, Map4k1, Matk, Mboat2, Mcf2l, Mdh2, Mettl1, Mettl9, Mgat1, Mgl2, Mical2, Mink1, Mitf, Mllt3, Mmachc, Mmp19, Mpp6, Mrc1, Mrpl12, Mrpl15, Mrpl47, Mrps18b, Mrps25, Mrps26, Mrps28, Msx3, Mtch2, Mtg1, Mtx2, Myc, Myo1e, Nans, Ndufs7, Ndufs8, Ndufv2, Nek6, Nfya, Nif3l1, Noc4l, Nol6, Nrbf2, Nsun2, Nudcd2, Nudt19, Nudt2, Nup50, Nxn, Ocstamp, Oit3, Olfm1, Osgep, Oxct1, P2rx5, P2ry1, Pcdh7, Pcid2, Pcyox1l, Pdcd1lg2, Pde12, Pde4dip, Pdgfa, Pdia4, Pdlim1, Pdzd11, Pecam1, Pex16, Pex26, Phldb1, Pi4k2a, Pi4ka, Pigx, Pim3, Pip5k1c, Pitpnb, Pkp2, Pla2g12a, Pla2g5, Plekha3, Plekhf1, Plekhf2, Plekhg1, Plk2, Plk3, Plxdc2, Pmepa1, Polr2g, Pop4, Ppan, Pparg, Ppargc1b, Ppif, Ppp1r1a, Ppp1r3c, Prkcd, Prkcsh, Prps1, Psat1, Psma4, Psmc5, Ptger2, Ptgir, Ptgs1, Ptpre, Ptrf, Qdpr, Rab19, Rab38, Rab3il1, Rars, Rbms1, Rbp4, Rcl1, Rdh13, Retnla, Rhoj, Ripk3, Rnase2a, Rnase4, Rpn1, Rps6kc1, Rras2, Rrp12, Rrp8, Rrp9, Rrs1, Rtkn, Rtn4rl1, Ryr1, Sardh, Scd2, Scn3a, Sdccag8, Sema4b, Serpinb9b, Sestd1, Setd8, Sf3b4, Sft2d2, Sh3kbp1, Slc1a5, Slc25a13, Slc25a5, Slc29a2, Slc30a4, Slc35b1, Slc39a6, Slc6a9, Slc9a3r2, Slmo2, Smad2, Smap2, Smg5, Smyd5, Snap29, Snn, Snx5, Socs2, Sphk1, Spint1, Srm, St6galnac4, St8sia1, Stt3b, Stx6, Stxbp6, Suclg1, Supv3l1, Taf10, Tanc2, Tarbp2, Tbc1d16, Tbrg4, Tbxas1, Tes, Tex9, Tfrc, Thyn1, Tiam1, Ticam1, Timm10, Timm8a1, Tmcc2, Tmco4, Tmem126b, Tmem144, Tmem158, Tmem26, Tnfaip8, Tnfaip8l3, Tnfrsf12a, Tnk2, Tomm70a, Tpcn2, Traf5, Trap1, Trmt61a, Tspan5, Tsr1, Ttc27, Tuba8, Tubd1, Txndc11, Ubl3, Uqcrc1, Usp39, Vdr, Vegfc, Vps53, Vwf, Wdfy2, Wdr46, Wdr74, Wfs1, Wsb2, Wwc1, Xpnpep1, Yars, Yrdc, Zbtb46, Zfp101, Znrf2 |

**Table S9 Datasets of peripheral blood whole-gene expression profiles of 15 autoimmune diseases included in the RRA study**

| **Series** | **Diseases** | **Samples** | **Tissues** | **Platforms** |
| --- | --- | --- | --- | --- |
| GSE81622 | SLE | 30 SLE patients and 25 controls | PBMCs | GPL10558 |
| GSE65391 | SLE | 924 SLE patients and 72 controls | Whole blood | GPL10558 |
| GSE61635 | SLE | 79 SLE patients and 30 controls | Whole blood | GPL570 |
| GSE49454 | SLE | 157 SLE patients and 20 controls | Whole blood | GPL10558 |
| GSE45291 | RA | 493 RA patients and 20 controls | Whole blood | GPL13158 |
| GSE117769 | RA | 51 RA patients and 50 controls | Whole blood | GPL16791 |
| GSE93272 | RA | 65 RA patients and 35 controls | Whole blood | GPL570 |
| GSE15573 | RA | 18 RA cases and 15 controls | PBMCs | GPL6102 |
| GSE80060 | JIA | 104 JIA patients and 22 controls | Whole blood | GPL570 |
| GSE81259 | JIA | 47 JIA and 14 controls | PBMCs | GPL16791 |
| GSE66795 | Sjögren's syndrome | 131 Sjögren’s syndrome patients and 29 controls | Whole blood | GPL10558 |
| GSE84844 | Sjögren's syndrome | 30 Sjögren’s syndrome patients and 30 controls | Whole blood | GPL570 |
| GSE51092 | Sjögren's syndrome | 190 Sjögren’s syndrome patients and 32 controls | Whole blood | GPL6884 |
| GSE73754 | Ankylosing spondylitis | 52 Ankylosing spondylitis patients and 20 controls | Whole blood | GPL10558 |
| GSE117928 | Systemic sclerosis | 18 Systemic sclerosis patient and 19 controls | PBMCs | GPL14951 |

SLE, systemic lupus erythematosus; RA, rheumatoid arthritis; JIA, juvenile idiopathic arthritis
